# Supplementary material for: Exploring common mechanisms of adverse drug reactions and disease phenotypes through network-based analysis
Source: Cell Rep Methods. 2025 Feb 14;5(2):100990. doi: 10.1016/j.crmeth.2025.100990 (PMC11955268; doi:10.1016/j.crmeth.2025.100990)
Supplement: Document S1. Figures S1–S5 and Table S1 [file mmc1.pdf]

Cell Reports Methods, Volume *5*

Supplemental information

Exploring common mechanisms of adverse drug reactions and disease phenotypes through network-based analysis

Farzaneh Firoozbakht, Maria Louise Elkjaer, Diane E. Handy, Rui-Sheng Wang, Zoe Chervontseva, Matthias Rarey, Joseph Loscalzo, Jan Baumbach, and Olga Tsoy

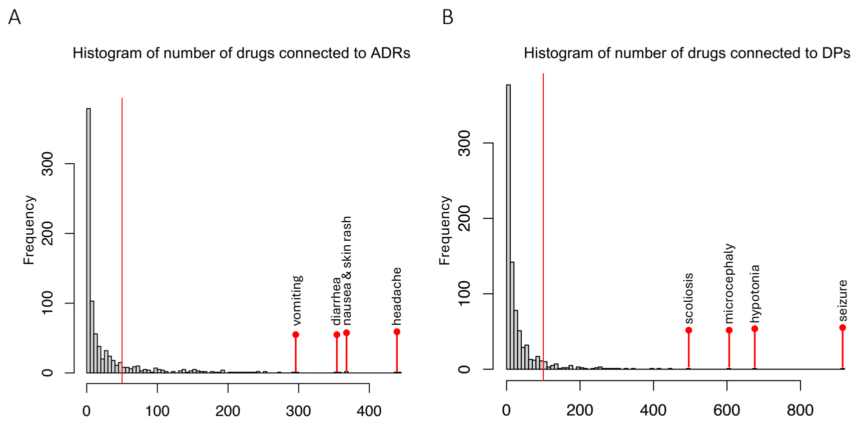

Figure S1. The distribution of the number of drugs and diseases connected to ADRs and DPs, related to STAR Methods.
(A) Number of drugs connected to ADRs. The vertical red line indicates the threshold used to remove ADRs connected to more than 50 drugs.
(B) Number of diseases connected to DPs. The vertical red line indicates the threshold used to remove DPs that are connected to more than100 diseases.

Table S1. The summary statistics of nodes and edges in our knowledge graph, related to STAR Methods.

Node/edge type	Initial count	# nodes &edges after preprocessing	Database
ADR	10368	649	MedDRA
DP	16874	649	HPO
Drug	6177	465	Drugbank
Disease	22825	2553	Mondo Disease Ontology
Protein	19385	12694	UniProt
ADR-[is the same as]-Phenotype	1200	649	BioPortal
ADR-[is reported for]-Drug	217384	6358	Sider
Drug-[has target]-Protein	26537	5185	DrugBank
Phenotype-[is reported for]- Disease	227812	9528	HPO
Gene-[associated with]-Disease	26841	6652	DisGeNET
Gene-[encoded by]-Protein	33246	3417	UniProt
Protein-[interacts with]-Protein	11938499	127767	STRING
Protein-[interacts with]-Protein	–	346535	Physical network

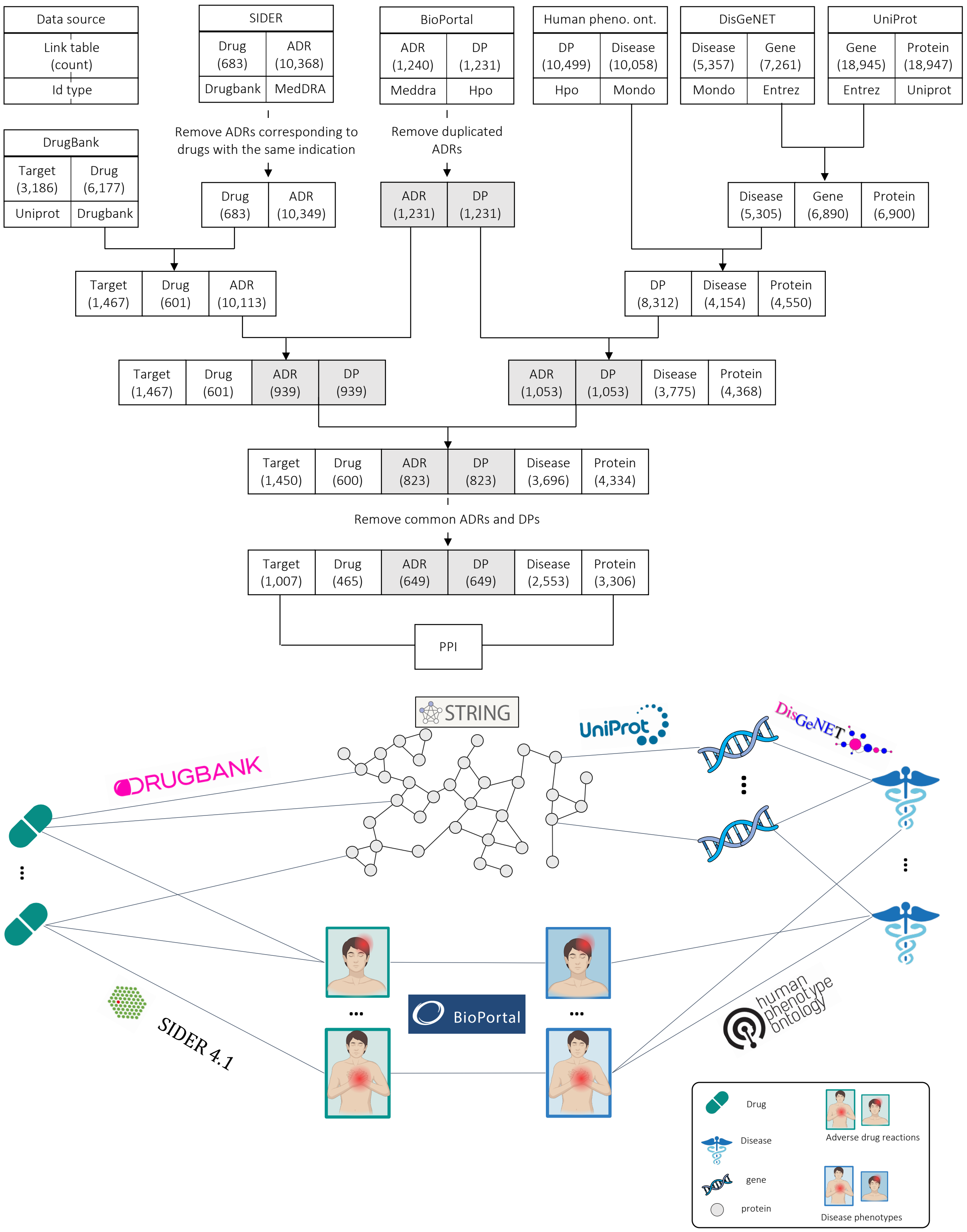

Figure S2. An overview of our knowledge graph, data sources and pre-processing, related to STAR Methods.

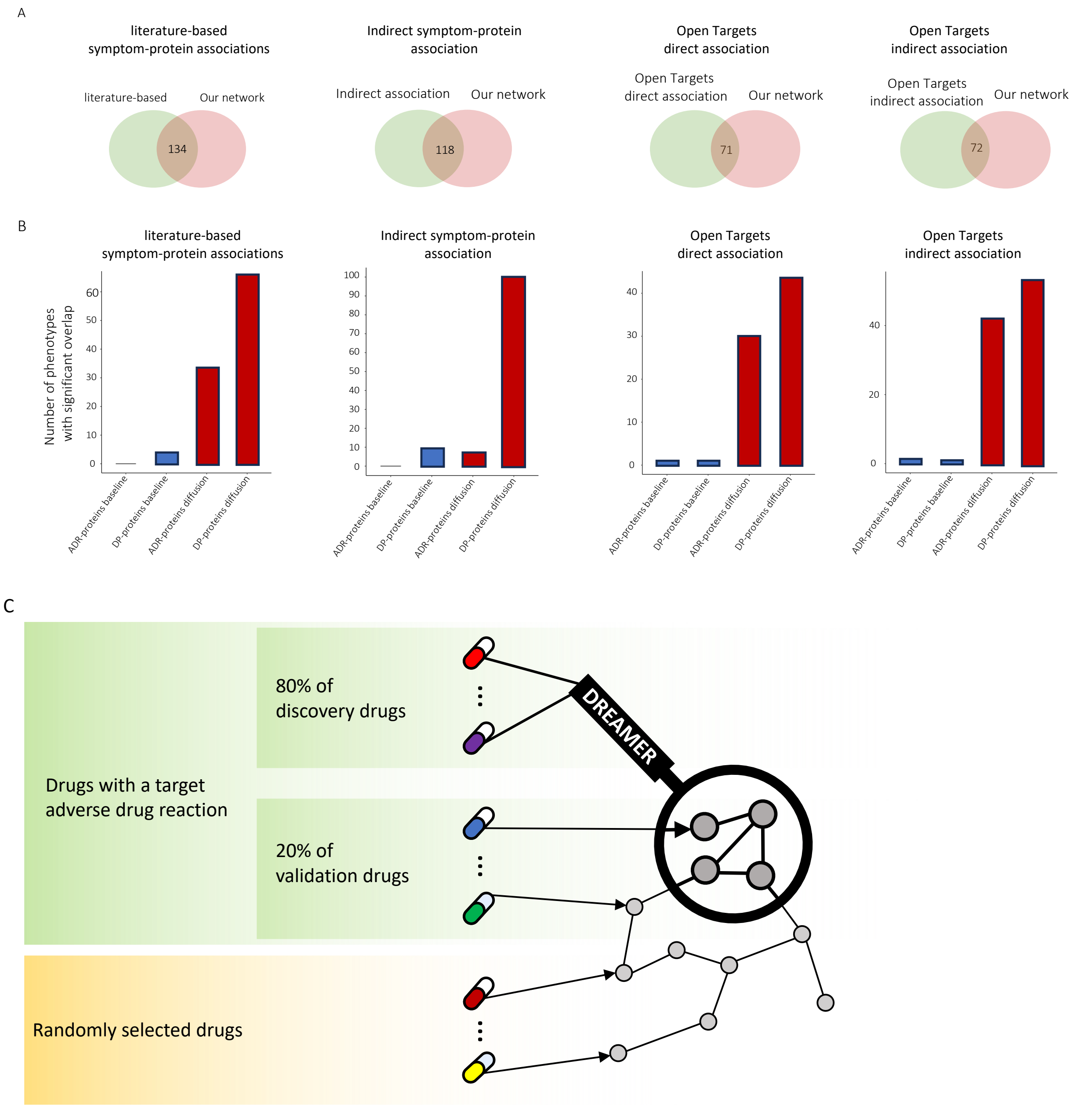

Figure S3. Reliability assessment of the identified protein set using the network diffusion algorithm on the physical protein-protein interaction network and schematic representing the hold-out validation analysis, related to Figure 2.

(A) Number of shared phenotypes with our constructed KG and known databases.

(B) Comparison of methods using literature-based dataset, indirect association based on disease-phenotypes equivalent terms, direct associations in Open Targets dataset, indirect associations in Open Targets dataset.

(C) Drugs with a particular ADR are split into a discovery set (80%) and a validation set (20%). The discovery drugs are used to identify ADR-DP proteins by the DREAMER pipeline. Our analysis shows that these ADR-DP proteins are closer to the validation drugs compared to randomly selected drugs that do not have the given ADR.

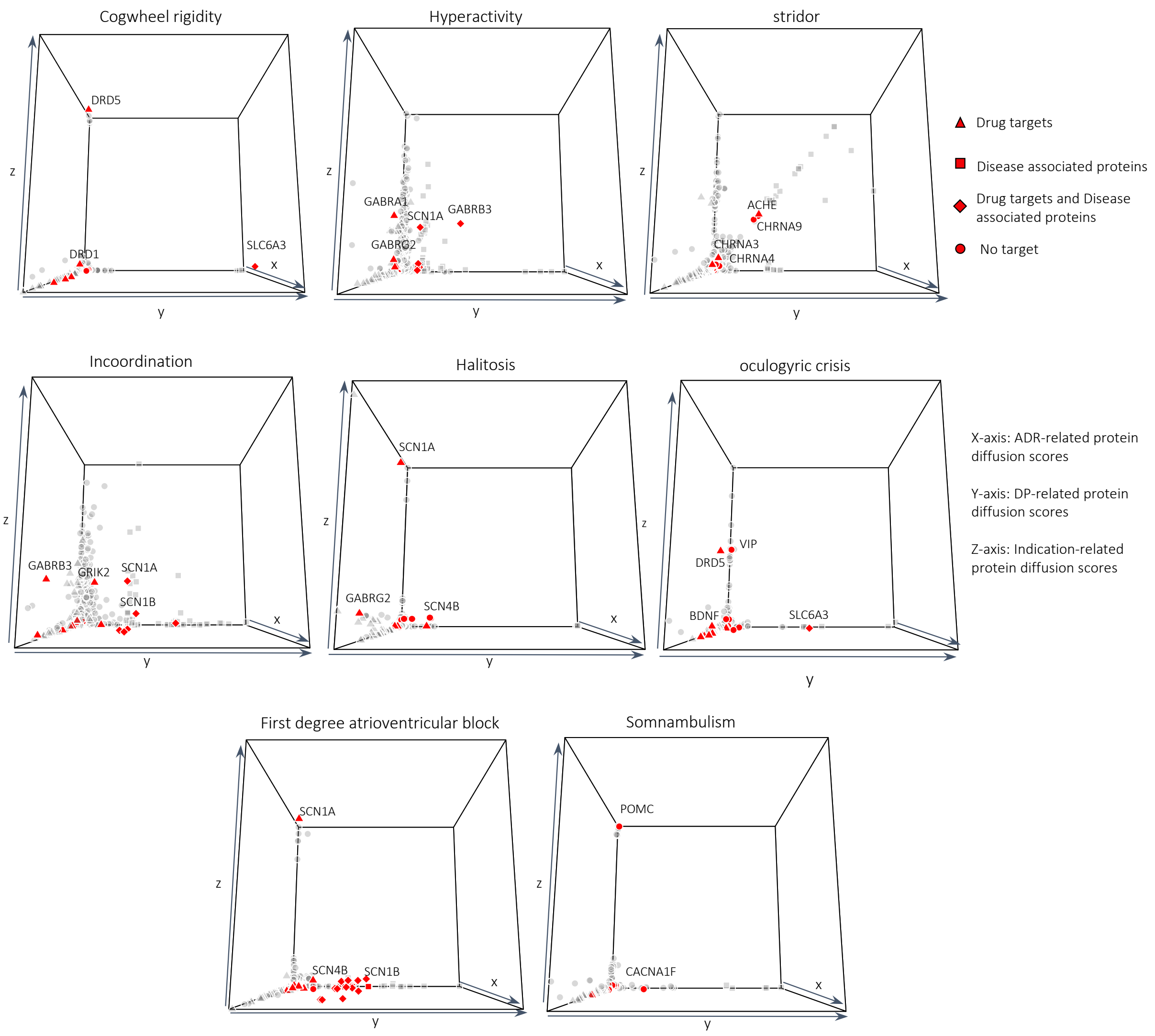

Figure S4. 3D diffusion maps, related to Figure 3. x, y, and z-axis represent the diffusion scores of proteins from drug-targets, disease-proteins, and drug indication-proteins, for cogwheel rigidity, hyperactivity, stridor, incoordination, halitosis, oculogyric crisis, first degree atrioventricular block, somnambulism. The ADR-DP proteins are indicated as red points.

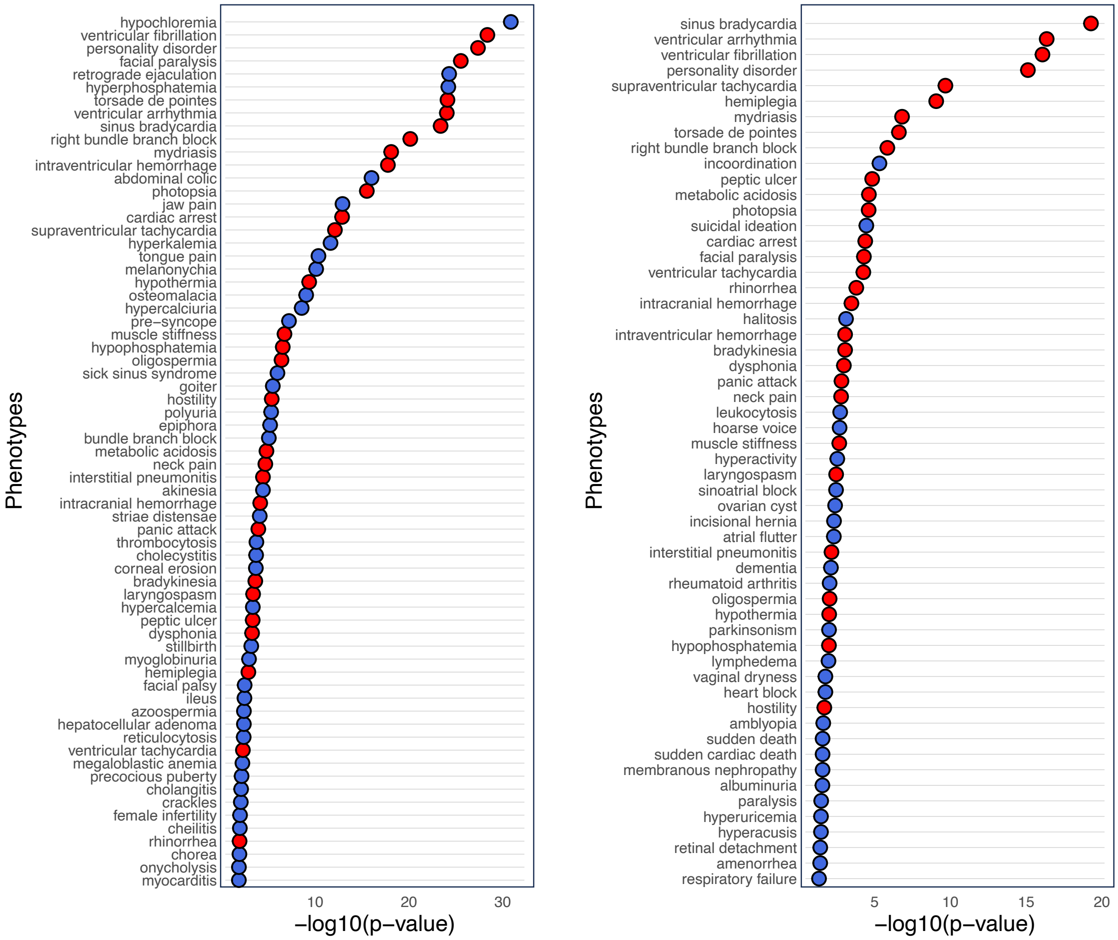

Figure S5. The ranked list of phenotypes based on the significance of their ADR-DP proteins, related to Figure 3. *Left*: for STRING network; *right*: for the physical PPI network. Phenotypes highlighted in “red” circles indicate those for which we identified the mechanism using an analysis based on either the STRING network or the physical PPI network.
