# Supplementary material for: Exploring common mechanisms of adverse drug reactions and disease phenotypes through network-based analysis
Source: Cell Rep Methods. 2025 Feb 14;5(2):100990. doi: 10.1016/j.crmeth.2025.100990 (PMC11955268; doi:10.1016/j.crmeth.2025.100990)
Supplement: Document S2. Article plus supplemental information [file mmc10.pdf]

Cell Reports
Methods

Article

Exploring common mechanisms of adverse drug reactions and disease phenotypes through network-based analysis

Graphical abstract

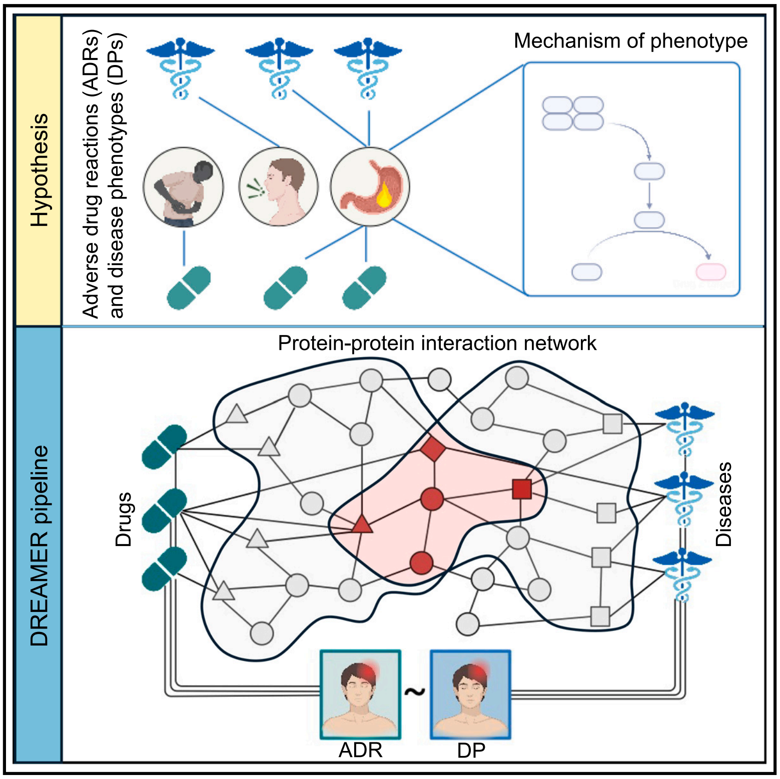

Highlights

- DREAMER is a network-based pipeline to explore the mechanism of clinical phenotypes
- Approach uncovers shared mechanisms of adverse drug reactions and disease phenotypes
- DREAMER provides insights for improving drug safety
- Potentially accelerates the process of drug repurposing

Authors

Farzaneh Firoozbakht,
Maria Louise Elkjaer, Diane E. Handy, ...,
Joseph Loscalzo, Jan Baumbach,
Olga Tsoy

Correspondence

farzaneh.firoozbakht@uni-hamburg.de

In brief

Firoozbakht et al. introduce DREAMER, a network-based framework that elucidates shared molecular mechanisms underlying adverse drug reactions and disease phenotypes. By integrating diverse biological data, DREAMER identifies proteins that reveal therapeutic insights, advancing drug safety and repurposing opportunities across various clinical contexts.

Firoozbakht et al., 2025, Cell Reports Methods 5, 100990
February 24, 2025 © 2025 The Author(s). Published by Elsevier Inc.
https://doi.org/10.1016/j.crmeth.2025.100990

CellPress

Cell Reports Methods

CellPress
OPEN ACCESS

Article

Exploring common mechanisms of adverse drug reactions and disease phenotypes through network-based analysis

Farzaneh Firoozbakht,[1,6,*] Maria Louise Elkjaer,[1] Diane E. Handy,[2] Rui-Sheng Wang,[2] Zoe Chervontseva,[1] Matthias Rarey,[3] Joseph Loscalzo,[2,5] Jan Baumbach,[1,4,5] and Olga Tsoy[1,5]
[1]Institute for Computational Systems Biology, University of Hamburg, Albert-Einstein-Ring 8-10, 22761 Hamburg, Germany
[2]Department of Medicine, Brigham and Women's Hospital, Harvard Medical School, Boston, MA, USA
[3]ZBH - Center for Bioinformatics, University of Hamburg, Hamburg, Germany
[4]Department of Mathematics and Computer Science, University of Southern Denmark, 5000 Odense, Denmark
[5]These authors contributed equally
[6]Lead contact
*Correspondence: farzaneh.firoozbakht@uni-hamburg.de
https://doi.org/10.1016/j.crmeth.2025.100990

MOTIVATION Adverse drug reactions (ADRs) are a major concern in healthcare and drug development, often resulting in severe clinical outcomes and leading to drug withdrawals. Despite their impact, understanding the complex, multifactorial nature of ADR mechanisms remains challenging, leaving critical gaps in drug safety assessments. An important question is whether network-based approaches, emerging as a promising frontier in systems pharmacology, can help uncover these mechanisms by integrating diverse biological and pharmacological data. We explored this question and developed DREAMER, a network-based framework designed to elucidate the molecular pathways underlying ADRs, with the aim of enhancing drug safety and identifying new opportunities for drug repurposing.

SUMMARY

The need for a deeper understanding of adverse drug reaction (ADR) mechanisms is vital for improving drug safety and repurposing. This study introduces Drug Adverse Reaction Mechanism Explainer (DREAMER), a network-based framework that uses a comprehensive knowledge graph to uncover molecular mechanisms underlying ADRs and disease phenotypes. By examining shared phenotypes of drugs and diseases and their effects on protein-protein interaction networks, DREAMER identifies proteins linked to ADR mechanisms. Applied to 649 ADRs, DREAMER identified molecular mechanisms for 67 ADRs, including ventricular arrhythmia and metabolic acidosis, and emphasized pathways like *GABAergic signaling* and coagulation proteins in personality disorders and intracranial hemorrhage. We further demonstrate the application of DREAMER in drug repurposing and propose sotalol, ranolazine, and diltiazem as candidate drugs to be repurposed for cardiac arrest. In summary, DREAMER effectively detects molecular mechanisms underlying phenotypes, emphasizing the importance of network-based analyses with integrative data for enhancing drug safety and accelerating the discovery of novel therapeutic strategies.

INTRODUCTION

Adverse drug reactions (ADRs) are important concerns in pharmacology and healthcare. They are a leading cause of mortality and drug withdrawals.[1] Gaining a deeper understanding of ADRs is essential for enhancing drug safety profiles and making informed healthcare decisions as they can reveal the complexity of *in vivo* human phenotypic responses.[2,3] By understanding the underlying mechanisms of ADRs, we can gain insight into a drug's mechanism of action, which can assist in identifying new drug targets, enhancing drug repurposing, predicting new therapeutic indications, and advancing personalized medicine.

Although some ADRs cannot be explained by known pharmacology and may result from non-specific interactions of reactive metabolites, drug kinetics, and/or environmental exposures, most ADRs are caused by unintended consequences of on-target or off-target drug-protein interactions.[4–7] Thus, drug-target interactions serve as valuable resources for understanding ADR mechanisms. Previous studies have considered the comprehensive set of drug targets to identify specific proteins

This is an open access article under the CC BY license (http://creativecommons.org/licenses/by/4.0/).

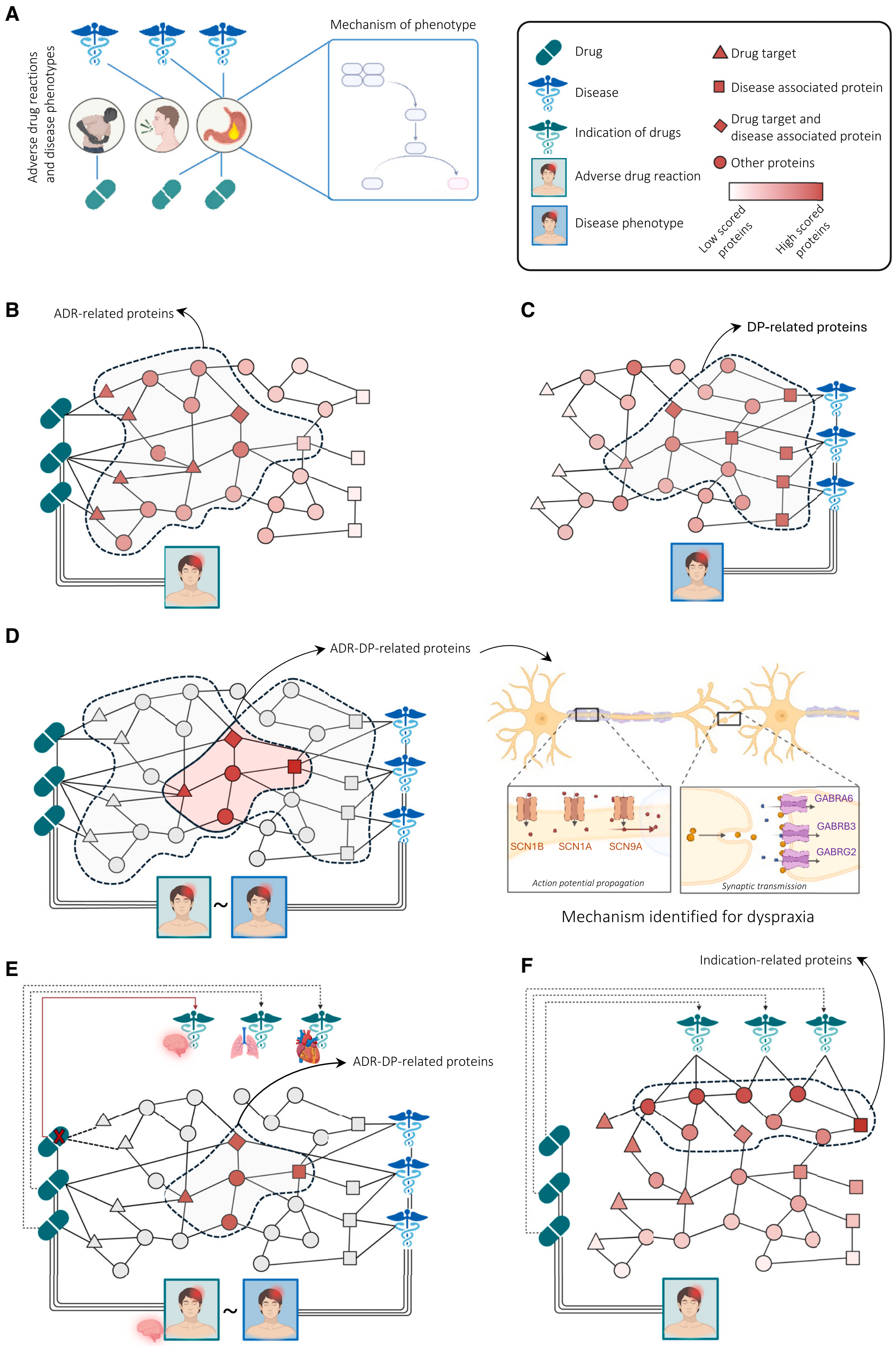

(legend on next page)

associated with ADRs. An initial computational method for identifying ADR-related pathways (i.e., biological pathways that can explain the mechanisms of an ADR) was developed by Wallach et al.[7] who hypothesized that drugs modulating the same pathways may lead to ADRs with similar phenotypes. To establish ADR-pathway relationships, they employed a logistic regression model to predict ADRs by quantifying drug-pathway interactions based on the docking scores of drugs to proteins within each pathway. Mizutani et al.[8] identified protein-associated ADRs by calculating the sparse canonical correlation between drug-protein relations and drug-side-effect relations. Kuhn et al.[9] further defined the relationship of proteins to ADRs by searching for statistically significant overlap between the set of drugs linked to their associated proteins and the set of drugs linked to the given ADR. To establish the relationship between ADRs and their potential drug targets, Lounkine et al.[6] calculated an enrichment score for each target-ADR pair based on their observed versus expected co-occurrence, and a statistical significance test was applied to find likely target-ADR associations. Lim et al.[10] constructed a heterogeneous network including drug, gene, and ADR nodes. They employed the ADR-gene pairs identified by Lounkine et al.[6] and applied a collaborative filtering-based algorithm to predict the missing links between ADRs and genes. Next, using a permutation-based algorithm, statistically significant genes for each ADR were ascertained and used for pathway enrichment analysis. Park et al.[11] assumed that ADRs reported for drugs targeting a single protein are entirely derived from perturbing that specific protein. Accordingly, they hypothesized that predicting the likelihood that a single-target drug causes an ADR corresponds to the probability that the protein target is associated with the ADR. Based on this concept, they reduced the problem of ADR-protein association prediction to the problem of drug-single-protein target prediction. To solve this problem, they constructed a network of drug-target and protein-protein interactions (PPIs) and used the node2vec representation algorithm to embed proteins and drugs into a low-dimensional vector space. They further used a logistic regression classifier for each ADR to score ADR-protein pairs.

Despite the importance of drug targets in understanding ADR mechanisms, relying solely on these targets can lead to false positives (or failure to identify the true causative pathways owing to a limited search space). In contrast, exhaustive human genetic research has identified numerous disease-related genes. These genes are often linked to disease phenotypes (DPs), which can be considered analogous to ADRs.[12] Such relationships can be leveraged to strengthen our confidence and reduce the false positives complicating the drug-target analysis approach. Nguyen et al.[13] hypothesized that phenotypes caused by genetic variations could predict those by drug interactions with the encoded proteins. They demonstrated a significant correlation between the organ systems affected by genetic variations and those exhibiting ADRs when targeting the encoded proteins.

Understanding the molecular mechanisms behind ADRs and DPs remains challenging. Existing approaches often treat ADRs and DPs separately, overlooking shared mechanisms. In this study, we introduce Drug Adverse Reaction Mechanism Explainer (DREAMER), a network-based method to uncover shared protein mechanisms between ADRs and DPs, which can enhance drug safety and repurposing[14] efforts. Specifically, we hypothesize that equivalent ADR-DP pairs, representing the same phenotype, arise from variations in shared biological pathways (Figure 1A).

To explore this hypothesis, we constructed a comprehensive knowledge graph (KG) integrating drugs, diseases, ADRs, DPs, and proteins. Our KG links drugs to ADRs and targets, and diseases to DPs and related proteins, and includes PPIs. DREAMER applies a network diffusion algorithm to identify proteins associated with ADRs and DPs, reducing potential false positives by integrating proteins linked to equivalent ADR-DP pairs. This dual perspective enables a holistic view of molecular mechanisms underlying shared phenotypes.

Key contributions of this study include the following:

(1) Constructing a KG that integrates diverse data sources, and particularly PPI networks, allowing analysis beyond individual proteins to capture broader molecular landscapes.
(2) Developing DREAMER, a network-based pipeline to identify proteins associated with clinical phenotypes.
(3) Providing a database of protein sets linked to phenotype mechanisms.

Overall, this study offers a systems-level perspective on joint ADR and DP mechanisms through DREAMER, integrating ADR- and DP-associated proteins to advance systems pharmacology and enhance our understanding of molecular mechanisms.

RESULTS

Dataset and network construction

We constructed a heterogeneous network, also referred to as a KG, where drugs, diseases, proteins, ADRs, and DPs are represented as nodes. Links between nodes were established using various databases, incorporating ADR-DP, drug-ADR, disease-DP, drug-target, disease-gene,[9,15–17] PPIs from STRING,[18] and

Figure 1. Overview of the DREAMER pipeline

(A) The basic hypothesis: phenotypically similar adverse drug reactions (ADRs) and disease phenotypes (DPs) might result from targeting of and variation in the same biological mechanisms and pathways.

(B) To obtain ADR-related proteins, we diffuse from the drug targets and perform a statistical test for each protein.

(C) To obtain DP-related proteins, we diffuse from the disease-related proteins and perform a statistical test for each protein.

(D) Left: ADR-DP proteins comprise the intersection set of proteins with significant overlap between ADR-related proteins and DP-related proteins; right: an example of identified ADR-DP proteins for dyspraxia (MedDRA: 10009696) phenotype.

(E) To limit potential confounding effects by organ/tissue-related indications, ADR-DP proteins are identified after removing the drugs with the same organ/tissue indication as the organ/tissue affected by the ADR.

(F) To analyze the confounding effects of drug indications, protein scores are determined by diffusing from proteins related to the indications of drugs associated with a specific ADR, resulting in the identification of significant proteins called indication-related proteins.

physical interactions.[19] Unless otherwise specified, results presented in the main text are based on the STRING network. An overview of our KG and the framework used for its construction are shown in Figure S2. Summary statistics and data sources for the network are provided in Table S1, with further details available in the STAR Methods section.

DREAMER pipeline

To explore the underlying mechanisms of a specific phenotype, DREAMER identifies proteins related to a pair of ADR and DP that exhibit the same phenotype. The step-by-step pipeline of DREAMER is summarized in the following:

(1) Identification of ADR-related proteins: we started by identifying proteins associated with the ADR. Using a network diffusion approach (i.e., personalized page rank; see STAR Methods), we diffused the signal from protein targets of drugs with a certain ADR over the PPI network. Therefore, as the initial condition for network diffusion, each protein in the network was assigned a probability score based on the frequency of being targeted by the drug associated with the queried ADR. This scoring process was followed by a permutation test (see STAR Methods), generating *p* values for each protein. We considered proteins with adjusted *p* values below 0.05 as significantly related to the ADR (Figure 1B).

(2) Identification of DP-related proteins: we applied the same network diffusion approach to identify proteins related to the DP with the same phenotype as the ADR in step 1, substituting drug targets with proteins related to the diseases linked to the queried DP. This step mirrors the ADR analysis and identifies DP-associated proteins (Figure 1C).

(3) Intersection to minimize false positives: to enhance the specificity of proteins identified for each queried phenotype, we obtained the intersection of the corresponding ADR-related (step 1) and DP-related (step 2) protein sets (Figure 1D). To evaluate the significance of the intersections, we applied the hypergeometric test, with Benjamini-Hochberg correction (adjusted $p < 0.05$). Proteins present in both sets that pass the significance test were designated as ADR-DP proteins, which we hypothesized to be involved in the mechanisms linking the ADR and DP. Among 649 phenotypes in our network, 120 of them showed significant overlap between their ADR-related and DP-related proteins. These 120 phenotypes with their identified proteins are listed in Tables S2 and S3.

(4) Considering the confounding effect of drug indications: we consider potential confounding effects related to drug indications by the following analysis:
   (a) Controlling the effect of indication-ADR organ overlap: removing drugs with the same organ/tissue indication as the ADR to avoid false associations (Figure 1E).
   (b) Controlling the effect of indication-related proteins: scoring proteins by diffusing from those related to drug indications to identify and remove significant indication-related proteins (Figure 1F).

After controlling for the confounding effect, the number of our significant phenotypes was reduced to 67 and their proteins are listed in Tables S4 and S5.

As an example of the ADR-DP proteins identified in our study, the phenotype dyspraxia (MedDRA: 10009696) was associated with six proteins: three sodium channels (SCN1A, SCN9A, SCN1B) for action potential propagation, and three $GABA_A$ receptor subunits (GABRB3, GABRG2, GABRA6) for synaptic transmission (Figures 1D; Table S2). $GABA_A$ receptors enhance sodium channel activation at myelinated axon nodes, regulating sensory feedback. Dysregulation can lead to dyspraxia due to impaired motor coordination.

As for visualization, we propose the diffusion map, which is a scatterplot representing each protein by its ADR-related and DP-related diffusion scores (Figure 2A). The red points show proteins with scores that are statistically significant for both ADRs and DPs and usually have large diffusion scores for both ADRs and DPs. It is worth mentioning that proteins with high diffusion scores might not necessarily be significant. In certain cases, these proteins may be hub proteins (i.e., highly connected), enhancing the probability of achieving high scores in the null model and leading to their rejection in the permutation test. As predicted, this analysis identifies many proteins involved in disease processes. Next, we provide three examples of protein sets identified by DREAMER as significantly associated with ventricular arrhythmia, vasculitis, and thrombocytosis.

For ventricular arrhythmia (Figure 2A), many of the significant proteins identified by the diffusion algorithm are ion channel proteins, such as those that contribute to Ca^{2+} (*CACNA1C*, *CACNA1C-IT2*), Na^+ (*SCN3A*, *SCN1B*, *SCN4B*, *SCN10A*), or K^+ (*KCNQ1*, *KCNE3*, *KCNH2*) transport in the heart.[20] Previously described mutations in *KCNQ1* and *KCNH2* are associated with dysfunction of the voltage-gated K^+ channel resulting in ventricular arrhythmias, such as long QT syndrome and ventricular fibrillation.[21] Additionally, patients treated for ventricular arrhythmias often have their potassium (K^+) levels tested and receive supplements if their levels are low. This is because hypokalemia, or low potassium levels, is a well-known risk factor for arrhythmias.[22] Mutations in Na^+-channel proteins (suprachiasmatic nucleus [SCN] proteins) can result in long QT syndrome or atrial fibrillation.[23] Some of these ion channels are also present in other tissues, including brain, muscle, stomach, and colon. For example, mutations in the *SCN1B* can increase not only the risk of cardiac arrhythmia but also epilepsy.[24] Therefore, drugs that alter their function can have cardiovascular, muscular, gastrointestinal, or neurological consequences, depending on which organs express the specific channels. Similarly, mutations in *CACNA1C* alter L-type voltage-gated Ca^{2+}-channels and are associated with long QT and short QT syndromes. An example is Timothy syndrome, the complex congenital syndrome caused by *CACNA1C* mutations,[25] which involves cardiac manifestations such as long QT, along with one or more non-cardiac phenotypes such as skeletal, facial, and neurodevelopmental abnormalities.[26]

Vasculitis encompasses a heterogeneous group of diseases involving large, medium, or small vessels depending on the underlying specific disease.[27,28] Hallmarks include damage or dysfunction of the endothelial cells that line blood vessels, and treatments vary depending on the specific type. The DP reflects the action of

CellPress
OPEN ACCESS

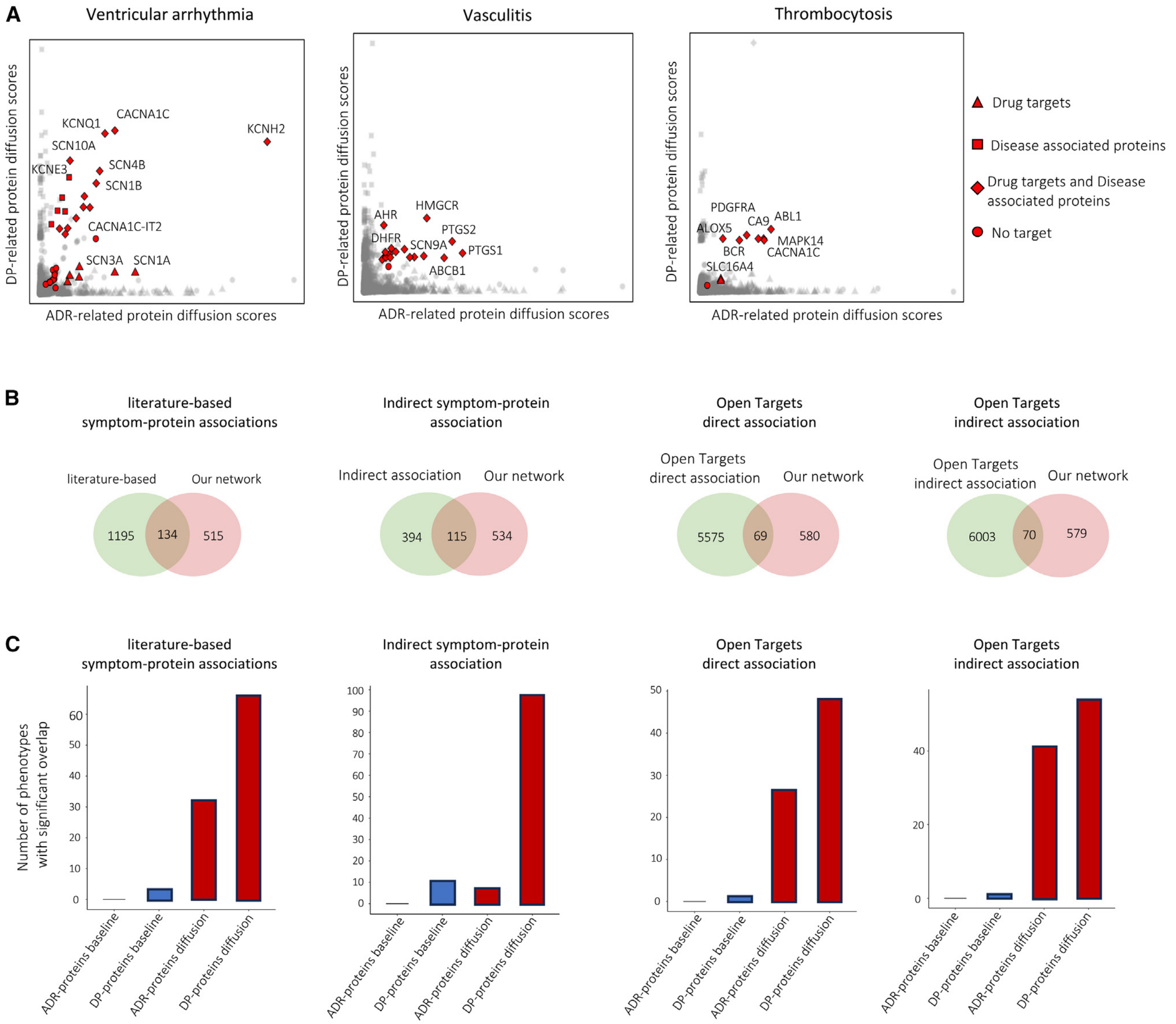

Figure 2. Diffusion map and reliability assessment of the identified protein set using the network diffusion algorithm on STRING

(A) Diffusion map for ventricular arrhythmia, vasculitis, and thrombocytosis. The abscissa and ordinate values represent the diffusion scores of proteins initiated from the drug targets and disease-associated proteins, respectively. In the diffusion map, drug targets are represented by triangles, disease-associated proteins by squares, proteins that are both drug targets and disease-associated proteins by diamonds, and proteins that are neither drug targets nor disease-associated proteins by circles.

(B) Number of phenotypes shared between our constructed KG and known databases

(C) Comparison of our diffusion-based method and the baseline method by identifying the number of ADRs and DPs with significant overlaps between proteins detected by different methods and those reported in the known databases (also see Figures S3A and S3B).

specific proteins that govern the inflammatory response (Figure 2A), including *PTGS1* and *PTGS2*, known as *COX-1* and *COX-2* enzymes. Kawasaki disease, a pediatric vasculitis, is treated with aspirin targeting these enzymes and thereby reducing inflammation. Similarly, methotrexate, which inhibits *DHFR*, is used in the treatment of other forms of vasculitis, having more potent anti-inflammatory effects than aspirin or non-steroidal anti-inflammatory drugs (NSAIDs). Activation of *AHR*, the aryl hydrocarbon receptor, is also associated with promoting vascular inflammation; however, downregulation of *AHR* can also exacerbate vascular injury by enhancing the function of monocytes and macrophages.[29,30] Statins, widely known for their ability to decrease cholesterol and reduce atherosclerosis via the inhibition of *HMGCR*, have beneficial anti-inflammatory effects on endothelial function and are being considered as additional therapies in some forms of vasculitis.[31,32]

Distinct from vasculitis, which involves inflammation of blood vessels, thrombocytosis is characterized by an elevated platelet

count. This hematologic abnormality is reflected in the identified proteins that drive the phenotype, such as platelet-derived growth factor receptor-alpha and beta (*PDGFR-*α and -β) (Figure 2A), which are present on both platelets and megakaryocytes, platelet precursors.[33] Inhibition of these tyrosine kinases with imatinib and other related targeted therapies reduces megakaryocyte survival and proliferation and decreases platelet numbers by blocking *PDGF* signaling. Myeloproliferative syndromes, including essential thrombocythemia, can result from mutations in the *JAK2*, *CALR*, and *MPL* genes, each acting as drivers of the fusion protein *BCR-ABL1* to increase cell (platelet as well as leukocyte) production.[34]

In summary, these examples demonstrate how the DREAMER pipeline can identify proteins that are mechanistically known to be associated with specific phenotypes.

Reliability assessment of the network diffusion method

In this section, we assess the reliability of our network diffusion algorithms in identifying relevant proteins compared to a baseline method. To do so, we examined the overlap between proteins identified by the algorithms (network diffusion-based and baseline) and proteins previously reported in the literature as associated with specific phenotypes (referred to as *a priori* known proteins). Specifically, we calculated how many phenotypes exhibit a significant overlap between ADR-related (or DP-related) proteins identified by our network diffusion-based method and known proteins. Significance was determined using a hypergeometric test with Benjamini-Hochberg correction (adjusted p <0.05). We then compared this count of significant phenotype overlaps with those observed using the baseline method. As a baseline, we implemented a published method[9] that links proteins to phenotypes based on statistical testing (see STAR Methods). Notably, both methods were applied to the same KG that we constructed for this study.

Although curated resources of *a priori* known proteins for ADRs and DPs are limited, they can still serve as valuable benchmarks for assessing the reliability of our identified proteins. Accordingly, we compiled known proteins from various available sources to use as a reference standard, as described in the following.

Literature-based associations

Lu et al.[35] compiled DP-related proteins from the PubMed and SemMed databases using natural language-processing methods and manual curation, identifying co-occurrences of DP and protein keywords in abstracts published before January 2022. In our analysis, we identified 134 phenotypes shared between the dataset of Lu et al.[35] and our dataset (Figures 2B and S3A). We evaluated the performance of our network diffusion method against the baseline by calculating the number of DPs whose identified proteins significantly overlapping literature-based proteins (Figures 2C and S3B). As shown in Figure 2H, our method achieved a significant overlap for 66 DPs, outperforming the baseline. Notably, we also observed a substantial overlap between our ADR-related proteins and literature-based proteins, despite the latter not covering ADRs.

Indirect associations

Several DP terms are equivalent to disease terms, allowing genes associated with these diseases to serve as *a priori* known proteins. We term these “indirect associations.” This equivalence enables evaluation of our identified DP by their overlap with proteins encoded by disease-related genes. Lu et al.[35] compiled a set of such indirect DP-gene associations using phenotype-genotype databases. We identified 115 DPs common to both our dataset and theirs (Figures 2B and S3A). As shown in Figures 2C and S3B, our identified DP proteins exhibit a significantly greater overlap with these indirect DP-gene associations compared to the baseline.

Open Targets-derived data

The Open Targets platform aggregates direct and indirect associations between targets and diseases from various sources, including genetic associations, somatic mutations, drugs, pathways, RNA expression, text mining, and animal models.[36] Direct associations are based on evidence explicitly linking a target to a phenotype. Indirect associations leverage the hierarchical structure of the disease ontology.

We identified 69 phenotypes with direct associations and 70 with indirect associations shared between Open Targets and our dataset (Figures 2B and S3A). As shown in Figures 2C and S3B, our method outperformed the baseline in identifying known proteins reported in the Open Targets dataset. Notably, none of the disease-gene associations in the Open Targets dataset was found in our KG.

While our diffusion-based model outperformed the baseline in identifying ADR- and DP-related proteins, we do not expect perfect overlap with known proteins. This is because the available known proteins are neither sufficiently comprehensive nor adequate as a complete ground truth. Therefore, although showing some degree of overlap between the identified ADR-related and DP-related proteins with proteins derived from *a priori* known proteins is useful for validation, we expect to recognize *de novo* proteins for each phenotype. Moreover, to reduce the false positives, we identified proteins present in both the ADR-related and DP-related sets for each phenotype, termed as ADR-DP proteins, as described in step 3 in DREAMER pipeline.

Holdout validation

DREAMER identifies proteins mechanistically related to specific phenotypes by analyzing the network proximity of proteins to drugs and diseases associated with that phenotype. This raises a question about the generalizability of DREAMER-identified proteins: would ADR-DP proteins remain relevant when our KG encounters new drugs and diseases? Accordingly, we hypothesized that any new drug and disease linked to a given phenotype would likely have at least one associated protein in closer proximity to the DREAMER-identified proteins than drugs and diseases without that phenotype (Figure S3C).

To test this hypothesis, we performed a holdout analysis to further validate our pipeline. For each phenotype, we split its associated drugs and diseases into two sets: 80% as the discovery set and 20% as the validation set. Additionally, for each phenotype, we randomly selected drugs and diseases that are not associated with the given phenotype, with an equal number to that the validation set. Here, we refer to the drugs and diseases in the validation set as “positive assets” and the randomly selected ones as “negative assets.”

In the discovery phase, we use DREAMER to identify proteins related to each phenotype based on the drugs and diseases in

Table 1. The *p* values obtained from Fisher's exact test for the holdout validation

Holdout type	Thresholds X = 0	X = 1	X = 2	X = 3	X = 4	X = 5	X = 6
Drug holdout	5.7e−08	1.4e−08	0.07	0.1	–	–	–
Disease holdout	3.1e−09	1.2e−10	2.7e−07	0.1	0.03	0.3	0.5
Drug holdout (after drug clustering)	0.003	0.003	0.9	0.9	0.9	–	–

the discovery set. In the validation phase, for each phenotype, we assessed the network proximity of the identified proteins with the positive and negative assets. We expect that proteins identified in the discovery phase for a given phenotype would show higher network proximity to the positive assets than to the negative assets. To assess the network proximities, we used the shortest path in the network.

Specifically, we counted the shortest paths of lengths less than or equal to $X \in \{0, 1, 2, \ldots\}$ for both positive and negative assets across all phenotypes. Using Fisher's exact test, we evaluated whether the proportion of positive assets with a shortest path $\leq X$ was significantly higher than that for negative assets. The results, shown in Table 1, indicate that positive assets are indeed significantly closer to DREAMER-identified proteins than negative assets. This finding supports our hypothesis that ADRs and DPs that exhibit the same phenotype can arise from variation in the same proteins and pathways.

Additionally, to ensure a more rigorous assessment, we repeated the process above by splitting the drugs into discovery and validation sets based on their molecular dissimilarities using the DataSAIL package in Python.[37] Specifically, DataSAIL employs an algorithm to minimize similarity between molecules in the discovery and validation set. To measure drug similarities, DataSAIL calculates Tanimoto coefficients between molecular fingerprints derived from their Simplified Molecular Input Line Entry System (SMILES) representations. This approach reduces the risk of information leakage and structural similarity between discovery and validation sets. The results are presented in Table 1 and are consistent with those from the previous holdout validation analysis.

Considering the confounding effect of drug indications

The proteins identified by DREAMER for a specific phenotype may have been recognized under the influence of the indications of the drugs with the corresponding ADR. In this context, we consider two types of potential confounding effects:

(1) Organ/tissue overlap: when a drug is used to treat a condition in a specific organ/tissue, it might also have targets that seem relevant to its associated ADR affecting the same organ/tissue. However, these associations might only reflect the drug's intended action in that organ or tissue, rather than the ADR's underlying mechanism;
(2) Indication-related proteins: for drugs with a specific ADR, the conditions they treat may be associated with proteins that are closely related to our identified ADR-DP proteins. In such cases, the identified ADR-related proteins may be influenced by the drug's therapeutic indications rather than a direct mechanistic link to the ADR itself.

In the following, we focus on each of the mentioned confounding effects and describe the pipeline we employed to address them.

To reduce organ/tissue-related confounding, for each ADR, we excluded all of its associated drugs with indications affecting the same organ/tissue as the ADR (Figure 1E). For example, for the cardiovascular-related ADR-phenotype tachycardia, we excluded all drugs with tachycardia as their ADRs that also had at least one cardiovascular-related indication. Specifically, we manually identified the relevant organs/tissues for the 120 ADR-DPs (Table S6) and obtained organ indications for the drugs from a previous study.[13] Among 465 drugs in our network, 328 were listed in their dataset. As a result, the number of ADRs with at least one associated drug was reduced to 97 (see Tables S4 and S5).

We then reapplied the DREAMER pipeline to obtain a new set of proteins for each phenotype. For 90 out of 97 phenotypes, we observed a significant overlap between the proteins identified before and after the organ/tissue-based drug removal described above (tested via hypergeometric test, $p < 0.05$) listed in Tables S4 and S5. We note that, after the removal of the drugs with indications in the same organ/tissue as the ADRs, the average number of drugs was reduced to 6.6 from 14.2 for each ADR. While, on average, 30% of the drugs are excluded in this analysis, the results do not show a significant variation, which further indicates the robustness of our pipeline.

To address the potential confounding effect of indication-related proteins, we investigated whether ADR-DP proteins for each phenotype interact with proteins associated with therapeutic indications of drugs linked to the same phenotype. Using the network diffusion algorithm over the PPI network (Figure 1F; see STAR Methods), we computed diffusion scores for proteins based on their PPI adjacencies with drug indications, as previously described for identifying ADR and DP proteins. Of the 120 phenotypes analyzed, 95 were linked to at least one drug that has at least an indication with at least one associated gene in our dataset (listed in Tables S4 and S5). Limiting our analysis to these 95 phenotypes, we obtained diffusion scores from the indications of drugs linked to each ADR. Figure 3 shows the diffusion map, now incorporating a third dimension representing diffusion scores based on drug indications. After identifying the diffusion score of proteins with respect to the drug indications, we performed a permutation test (see STAR Methods) to assign a *p* value to each protein. Proteins with corrected $p < 0.05$ were considered as indication-related proteins (see STAR Methods). We then recognized phenotypes with significant overlap (hypergeometric test with Benjamini-Hochberg adjusted $p < 0.05$) between the indication-related proteins and ADR-DP proteins. For 84 out of 95 phenotypes (listed in

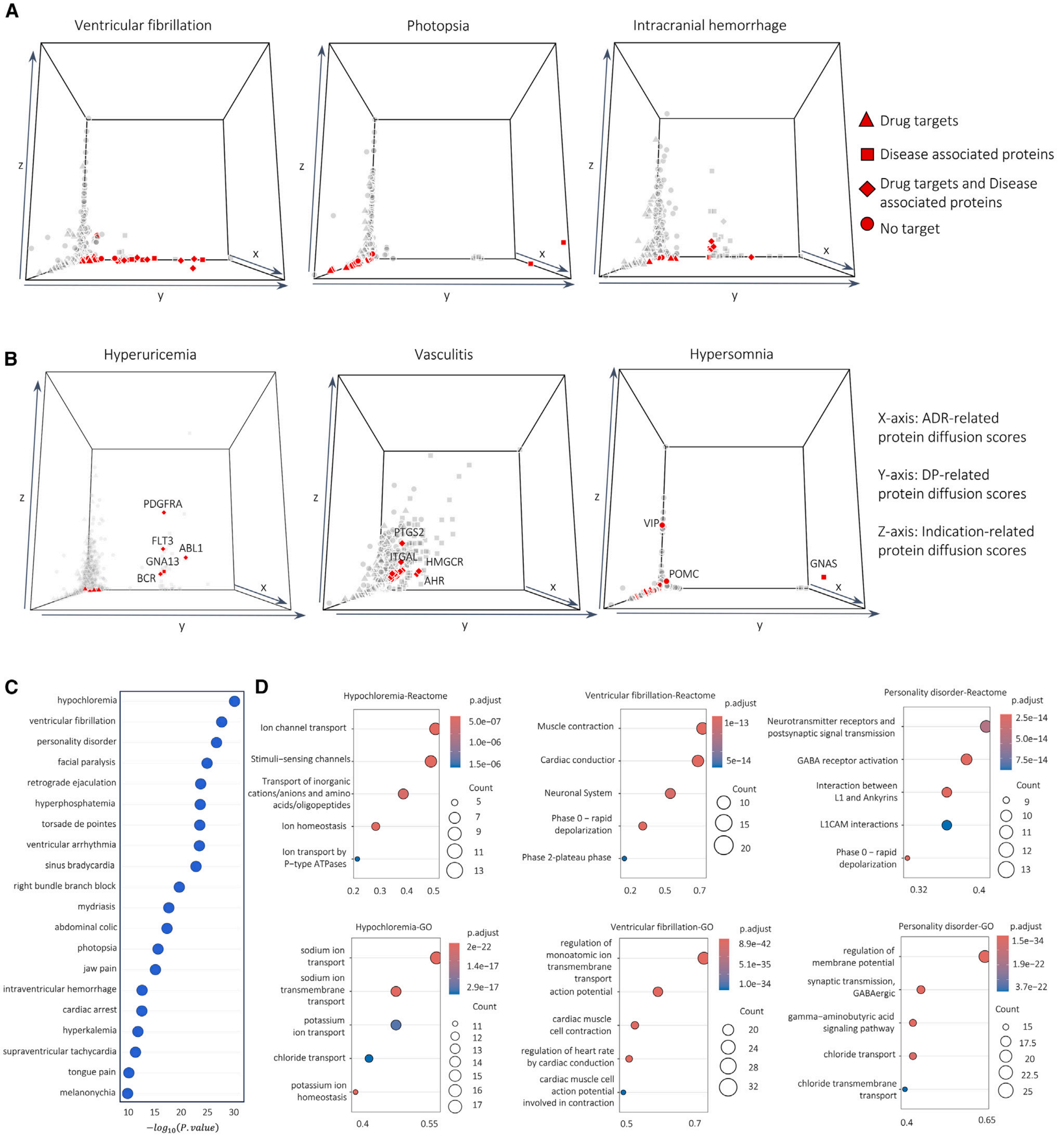

Figure 3. 3D diffusion map and pathway enrichment results for the top phenotypes

(A) Phenotypes whose identified ADR-DP proteins (indicated in red) are far from the subnetworks related to drug indications. In diffusion maps, the x, y, and z axes represent the diffusion scores of proteins from drug targets, disease-related proteins, and drug-indication-related proteins.

(B) Phenotypes whose identified ADR-DP proteins (indicated in red) have larger values in the third axis, and their independence from indication-related proteins is not trivial (also see Figure S4).

(C) The ranked list of phenotypes based on the significance of their ADR-DP proteins.

(D) Reactome and Gene Ontology over-representation analysis for ADR-DP proteins of the top three phenotypes.

Tables S4 and S5), no evidence of significant overlap was found. As can be seen in Figure 3A, in these phenotypes, the ADR-DP proteins (indicated in red) have smaller diffusion scores with respect to the third dimension, suggesting that, for these phenotypes, the subnetworks related to drug indications are far from those related to ADR-DP proteins. For example, intracranial hemorrhage, a critical condition involving bleeding within the brain, was linked to proteins of the coagulation and anticoagulation pathways such as protein C) PROC), factor X (F10), and prothrombin (F2) (Table S2). Dysregulation of these proteins can impair hemostasis and formation of stable clots, leading to an increased risk of excessive bleeding events such as intracranial hemorrhage. These findings suggest that, at least for these phenotypes, the identified ADR-DP proteins have no significant association with the protein drivers of their clinical indications for their related drugs.

By contrast, for the remaining 11 phenotypes (Figure 3B; Figure S4), the ADR-DP proteins show higher values on the third axis, indicating a non-trivial independence with indication-related proteins that requires a further investigation by domain experts.

Accordingly, the 3D diffusion map can help to inspect such ambiguities. Put another way, high z axis values may reflect reverse causality, where the ADR is a downstream consequence of treatment and not directly associated with the underlying phenotype mechanism. For example, in hyperuricemia, we identified ADR-DP proteins that included *PDGFRA*, *FLT3*, and *ABL1* as having high values in the z axis. These proteins are commonly targeted by drugs for cancer treatment, including leukemia, and play roles in the differentiation, division, and growth of cells. Cell death induced by these treatments can lead to great increases in uric acid in the blood, overwhelming the body's normal ability to clear that metabolite and ultimately causing renal dysfunction, further worsening the hyperuricemia. In vasculitis, proteins like *PTGS2* (Figure 2B) are crucial in mediating inflammation via prostaglandin synthesis. Incorporating indication-related proteins, we observe an overlap in inflammatory activation between the indication (z axis) and vasculitis (y axis) (Figure 3B). Similarly, *ITGAL* (*CD11a/LFA-1*), essential in leukocyte migration,[38] may be detected due to the (1) modulation of immune filtration by the indicated drug/disease and/or (2) vasculitis itself affecting immune cell-endothelial interactions. For hypersomnia, *VIP*'s high z axis value may relate to *VIP*omas, where octreotide inhibits excessive *VIP* secretion. However, *VIP* is produced by neurons in the *SCN* of the hypothalamus where it maintains normal circadian rhythms,[39] supporting its potential involvement in hypersomnia too. *VIP*'s role in various phenotypes likely depends on its anatomic location and quantification.

According to both analyses discussed above, we identified mechanisms for 67 phenotypes that show no evidence of association with drug indications (Table S4). Additionally, when replacing the STRING PPI network with the physical PPI network, our analysis identified mechanisms for 56 phenotypes (Table S5) that are not related to drug indications. Notably, there was an overlap of 29 phenotypes between these two analyses. The ADR-DP proteins identified from both analyses for all these 29 phenotypes showed significant overlap (hypergeometric test, adjusted using the Benjamini-Hochberg method, $p < 0.05$).

Biological insights into phenotype mechanism of action

In this section, we investigate the biological function of the protein sets identified by DREAMER for 67 phenotypes with no evidence of association to drug indications, based on evidence from pathway enrichment analysis and supported by prior literature to connect these findings to known physiological and pathological processes, while knowing limitations of indirect connections. We first ranked these phenotypes based on the significance of the overlaps between their ADR proteins and DP proteins, which supports our hypothesis that overlapping ADR and PD protein sets may indicate shared underlying biological mechanisms. Figure S5 shows the ranking of all phenotypes, with the top 20 phenotypes shown in Figure 3C. We then found the enriched pathways for the ADR-DP protein sets based on an over-representation analysis using the Reactome[40] and Gene Ontology[41] (Table S7) databases. The results for the top three ranked phenotypes are illustrated in Figure 3D.

Pathway analysis highlights the physiological processes involved in these disorders. For example, chloride is an anion that is mostly found in the extracellular space. Its concentration is regulated by the gastrointestinal tract, where it is absorbed from food, as well as the kidney, where it is excreted in urine or reabsorbed in the proximal tubule. Chloride transport relies on transmembrane ion transporters and cotransporters as well as additional Na^+/K^+ ATP-dependent ion transporters that provide energetics for Cl^- transport (Figure 3D). Thus, its concentration is dependent on that of other ions, such as Na^+, K^+, and bicarbonate (HCO_3^-). Owing to its inverse relationship with bicarbonate, hypochloremia can result in metabolic alkalosis. Hypochloremia can occur due to gastrointestinal causes, such as vomiting; or renal loss of chloride due to the use of diuretics (hypochloremic metabolic alkalosis due to excessive fluid loss leading to volume contraction); and/or because of hyponatremia and hypokalemia, as the fluxes in sodium and potassium will affect chloride levels.[42]

The coordinated movement of ions through voltage-gated ion channels is important to maintain the rhythmic beating of the heart (Figure 3D). Disruption of these processes leads to abnormal action potentials, arrhythmias, and ventricular fibrillation. Many of these ion channels also play a role in other organs, including the brain.[43]

In personality disorder (a complex, comparatively non-specific phenotype), the identified pathways are all known key mechanisms for various psychiatric conditions. The neurotransmitter receptors and postsynaptic signal transmission reflect the significant roles of dysregulated neurotransmitter systems implicated in a range of personality disorders such as mood and bipolar disorder. Altered phase 0, representing rapid neuronal depolarization, can lead to epilepsy[44] (Figure 3D). Dysregulated membrane potential can be influenced by chloride transport and can impair GABAergic transmission (Figure 3D). Impairment in GABAergic transmission plays a significant role in the pathophysiology of major depressive disorder (MDD),[45] schizophrenia,[46] bipolar disorder,[47] and autism,[48] and lower levels of GABA are often identified as the main endophenotype of

MDD.[49] The antidepressant effect of ketamine may also be related to its selective impact on GABAergic interneurons, blocking NMDA receptors and reducing inhibitory signals to enhance cortical excitation. Additionally, the interaction between L1CAM and ankyrins (Figure 3D) guides neuronal adhesion and signaling, where abnormalities are associated with neurodevelopmental disorders like autism.[50] These mechanistic phenotype pathways emphasize the interconnected roles of neurotransmitter signaling, synaptic function, and neuronal excitability in personality (and other psychiatric) disorders.

Overall, these results provide preliminary insights into potential biological mechanisms connecting ADRs and DPs through shared protein pathways. We note that, while pathway enrichment and literature evidence support these findings, future experimental studies need to confirm these mechanistic connections.

Application of DREAMER for therapeutic potential

The proteins identified for each phenotype using DREAMER can open new avenues for drug design and drug repurposing (i.e., an approach to identifying new therapeutic uses for drugs that are already approved for specific disorders).[14] It can be hypothesized that targeting proteins identified for each phenotype is most likely either to induce or treat the phenotype, as one cannot determine directionality *a priori* from this analysis. Therefore, DREAMER can be leveraged for drug discovery in two ways: (1) predict possible ADRs for new drugs based on their known targets, and (2) design new drugs or suggest repurposing candidates, based on their targets, for a disease.

In particular, to showcase the application of DREAMER for drug repurposing in the context of the second case, we focus on phenotypes for which there is evidence that targeting their ADR-DP proteins can treat the corresponding phenotype. For this purpose, we identified phenotypes whose ADR-DP proteins contain at least one protein targeted by a drug with an indication with the same terminology as the specified ADR. For example, cardiac arrest is a terminology that is assigned to an ADR (MedDRA: 10007515 in the SIDER dataset), a DP (with hpo:0001695 in the Human Phenotype Ontology [HPO] dataset), and drug indication (with mondo:0000745 in Mondo Disease Ontology dataset). Interestingly, we found three drugs (diltiazem, carvedilol, and verapamil) that are indicated for cardiac arrest and target at least one of the proteins recognized by DREAMER for cardiac arrest (Figure 4). Thus, hereafter we refer to such drugs as "indicated drugs." In our dataset, we identified a total of eight such phenotypes, namely cardiac arrest, hypophosphatemia, precocious puberty, Torsades de Pointes, thrombocytosis, peptic ulcer, ventricular tachycardia, and ventricular fibrillation (Table S8). Figure 4 illustrates PPI subnetworks for five of those phenotypes restricted to their ADR-DP proteins along with the drugs that target them and have indications for those phenotypes. The complete list of the indicated drugs along with their targets among ADR-DP proteins is provided in Table S8.

To find opportunities for drug repurposing on the above-mentioned phenotypes, we found all the drugs that have at least one target among their ADR-DP proteins and focused only on those with no ADR on the corresponding phenotype and those that have not previously been found to have an indication for the corresponding phenotype in our dataset (Table S9). We, thus, refer to these drugs as candidate drugs for repurposing. For example, sotalol is recognized for its efficacy in treating various cardiac arrhythmias by targeting *KCNH2* (hERG) channels. In our dataset, sotalol is recognized to have indications for ventricular fibrillation. However, sotalol can cause prolongation of the QT interval, leading to ventricular arrhythmias such as ventricular tachycardia, ventricular fibrillation, cardiac arrest, and, in particular (based on our dataset confirmed by the published literature), Torsades de Pointes.[51–53] Although sotalol has not been reported to be related to cardiac arrest in our dataset, it is among the candidate drugs for the treatment of cardiac arrest in our analysis (Table S9). Similarly, ranolazine, a drug used to treat angina pectoris, has been used off-label for the treatment of ventricular arrhythmias.[54] In addition, we conducted a comprehensive search on the clinical trials website (ClinicalTrials.gov) to find evidence for these candidate drugs. Using a customized Python script, we queried all pairs of phenotypes and their candidate drugs and then carefully inspected all the derived results. Accordingly, we found a number of such phenotype-drug pairs listed in Table 2 along with their ClinicalTrials.gov IDs.

To repurpose novel drugs for these phenotypes, we scored and ranked all candidate drugs based on the ratio between the number of proteins they target within ADR-DP proteins and their overall number of targets. Figure 5 shows these rankings for the top 20 drugs, with circle sizes representing the total number of targets of each drug within the ADR-DP protein set. The circles are colored red to indicate drugs that have been found to be under investigation in clinical trials as being relevant to the queried phenotype. Candidate drugs that target proteins also targeted by indicated drugs may have a potential for repurposing (marked in green), as targeting these proteins has already been shown to be effective in treating the phenotype, but experimental testing is required to establish their efficacy. Circles colored in blue could potentially represent interesting findings to repurpose for their corresponding phenotypes, as they have not been previously targeted for the treatment of those phenotypes. Moreover, we hypothesize that drugs with higher ranks are more likely associated with the corresponding phenotype.

In summary, DREAMER provides a framework for identifying potential therapeutic targets; however, further research is essential to validate these findings. Future studies should focus on experimental and clinical validation of these candidate drugs and explore the mechanistic basis of their associations with the specified phenotypes.

DISCUSSION

In this study, we present DREAMER, a network-based method designed to investigate the underlying mechanisms of DPs and ADRs. While previous efforts have explored the mechanisms of ADRs and DPs separately, focusing on ADR-target PPIs or DP-gene associations, our approach integrates both phenotypes, offering a more comprehensive insight into their mechanisms. By identifying interconnected modules between ADRs and DPs, DREAMER effectively uncovers shared molecular mechanisms underlying 67 phenotypes, supporting our hypothesis.

CellPress
OPEN ACCESS

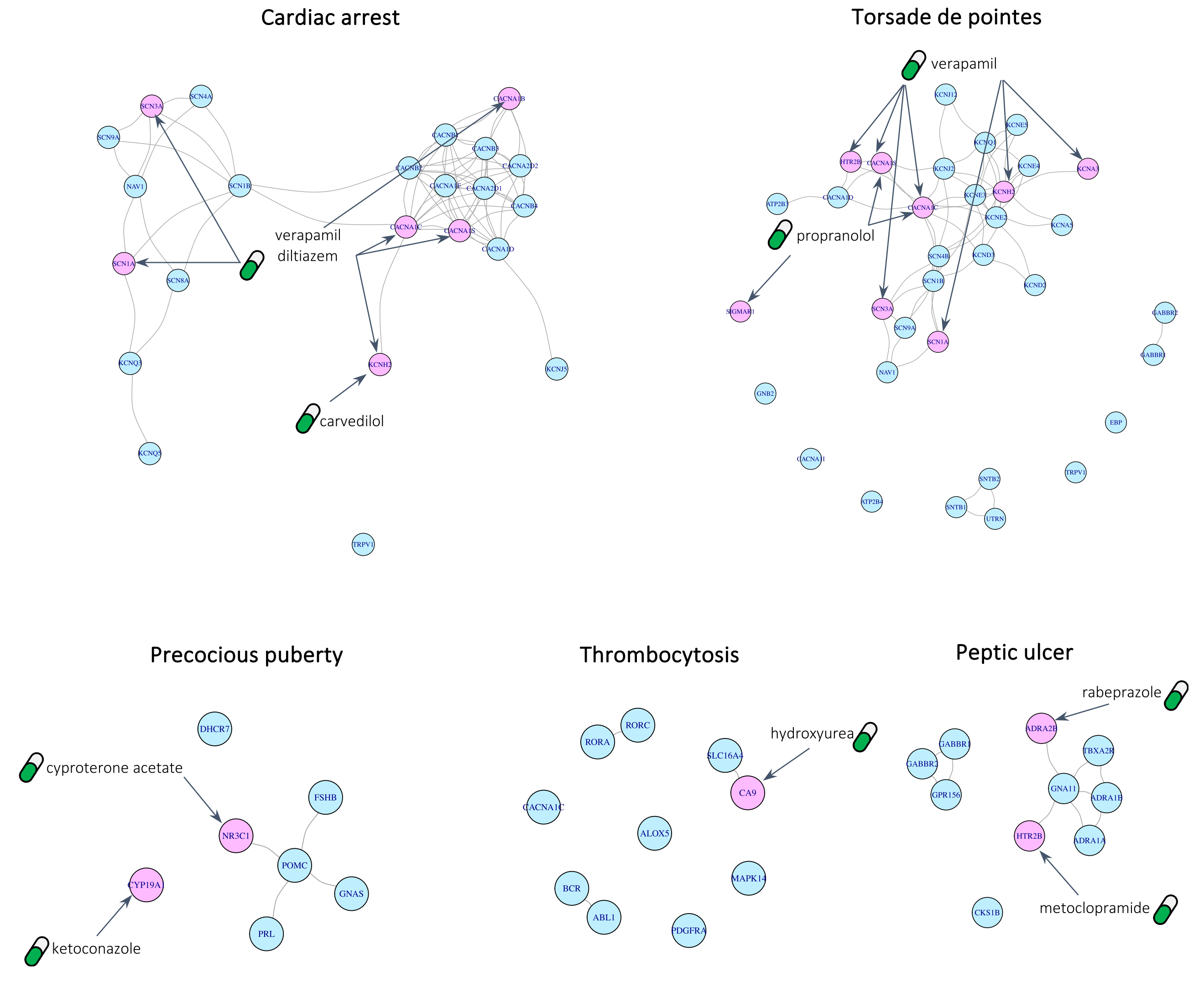

Figure 4. Subnetworks of identified protein sets along with indicated drugs that target them
Pink nodes are the proteins that are targeted by indicated drugs, and blue nodes are the rest of the ADR-DP proteins.

Furthermore, our reliability assessments and validation analyses confirm the robustness of DREAMER's approach, underscoring its contributions to systems pharmacology. Our pipeline has the potential to identify biomarkers for designing safer therapeutic strategies that minimize the need for drug discontinuation and enhance opportunities for drug repurposing, thereby supporting more effective and personalized treatments.

Network-based methods have proved to be effective in understanding the complex biology and systems pharmacology.[55–57] By representing biological entities as interconnected nodes in a network, these methods enable the identification of interactions and functional modules that may not be apparent when examining isolated entities. Unlike traditional approaches, which often rely on linear associations, network-based methods capture the interconnected nature of biological processes, providing a holistic view that can account for indirect and higher-order relationships across entities. This approach is particularly beneficial in exploring mechanisms of ADRs and DPs, as it allows us to understand how proteins and pathways implicated in therapeutic indications might overlap with or diverge from those contributing to adverse effects.

Targeting proteins in the organ of interest with drugs provides the basis for *in vivo* experiments that can explain the relationship between the functionality of that protein, systemic effects, and phenotypic responses. These proteins can be both on target and off target of drugs. While they are primarily targeted to treat specific indications or their phenotypes, they can also induce unintended side effects. Currently, drug safety evaluation, to a large degree, relies on animal experiments, which do not always translate reliably to humans owing to inherent biological differences.[12] In recent years, the increased availability of public databases including drug targets and ADRs has become a more reliable source of human-specific information. Genetic variations, by contrast, can be considered natural experiments, providing

Table 2. The phenotype-drug evidence for candidate drugs in the ClinicalTrials website

Phenotype	Candidate drug	ClinicalTrials.gov ID
Ventricular tachycardia	ranolazine	NCT01590979
Cardiac arrest	ranolazine, domperidone	NCT00998218, NCT04024865, NCT02500108, NCT01907633, NCT00925457
Peptic ulcer	baclofen	NCT00414856, NCT00461604, NCT00978016
Precocious puberty	anastrozole, letrozole	NCT00094328, NCT00055302
Torsades de Pointes	progesterone, testosterone	NCT01929083, NCT02513940
Ventricular fibrillation	ranolazine	NCT01887353, NCT01558830

insights into the mechanism of phenotypes. Genome-wide association studies have been extensively utilized to identify novel therapeutic targets, with a greater probability of drug approval when these targets are corroborated by human genetic evidence for the desired indication. Additionally, there is a growing interest in harnessing human genetic studies to predict the risk of ADRs. The importance of applying this strategy is more pronounced where suitable animal models for drug safety assessment are lacking.[12] Protein modules that are affected by both drugs with a specific ADR and diseases with a similar phenotype provide more evidence to explain the mechanism underlying the phenotype. Our study advances this concept by considering modules targeted by drugs and diseases exhibiting phenotypically similar ADRs and DPs.

The ADR-DP proteins identified by DREAMER may be influenced by the drug indications in the PPI, which can be recognized by their large values along the z axis in the 3D diffusion map (Figures 3A and 3B). Such instances may affect our interpretations and should be addressed carefully. Specifically, we encountered three scenarios: (1) ADR-DP proteins have an indirect association with the phenotype of interest, as seen in cases like hyperuricemia caused by cancer therapies; (2) higher-order relationships between the drug indications and the phenotype (such as both being related to the same tissue or organ); and (3) the phenotype mechanism stems from on-target effects. We acknowledge that, while the last scenario does not limit our interpretation, distinguishing it from the first two scenarios might be challenging or even infeasible.

Some phenotypes are multifactorial and are not directly linked to the drug's molecular effect(s) alone. However, it is still valuable to explore whether any of these phenotypes have a specific molecular mechanism that connects them to both ADRs and DPs simultaneously. For example, in the case of female infertility, which often stems from prior infections, DREAMER has identified proteins enriched in the metabolism of steroid hormone pathways (Tables S2 and S7). In contrast, dementia may arise from multiple factors beyond genetics, such as age, lifestyle, social engagement, and cognitive function, which would be discarded by DREAMER owing to the lack of significant overlapping proteins connecting the ADRs and DPs.

In this analysis, we used the STRING network, which integrates diverse PPIs from various sources, including direct physical interactions and indirect associations such as genetic co-occurrences, co-expression, and computational predictions. This broad interaction dataset enables exploration of phenotype mechanisms through higher-order interactions. While STRING offers a comprehensive view of generalized interaction data, we also applied DREAMER using a physical PPI network, which focuses exclusively on high-confidence interactions validated by experimental methods such as yeast two-hybrid screening, NMR spectroscopy, X-ray crystallography, and cryoelectron microscopy. Shared phenotypes between the STRING and physical PPI networks are extensively studied, commonly encountered in clinical practice, and span multiple organ systems, such as cardiac arrest, bradykinesia, ventricular arrhythmia, and interstitial pneumonitis (Figure S5). However, the physical PPI network uniquely identifies dementia and parkinsonism (cognitive and motor symptoms), while STRING uniquely identifies akinesia, a symptom of parkinsonism related to movement initiation difficulties. The reasons for these distinctions remain uncertain at this time but likely reflect the complexity of the phenotypes as well as their lack of specificity in some cases. Similar trends are observed in inflammatory phenotypes: both networks identify interstitial pneumonitis, but the physical PPI network uniquely captures systemic inflammatory conditions (e.g., rheumatoid arthritis, leukocytosis, involving direct interactions with mediators derived from circulating immune cells), while STRING identifies organ-specific inflammation (e.g., cholecystitis, cholangitis). Overall, STRING identifies more phenotypes and a broader range of conditions, including structural cardiac and metabolic abnormalities, reflecting its integrative approach. In contrast, the physical PPI network identifies disorders with better-characterized molecular mechanisms.

To assess the validity of our identified proteins, we conducted several analyses: (1) reliability assessment, to demonstrate the overlap of our identified diffusion-based proteins with *a priori* known proteins and show their superiority over a baseline model; (2) holdout validation, to show that the identified proteins are generalizable to new drugs and diseases that are added to our KG; and (3) robustness assessment, to demonstrate that the identified proteins statistically remain consistent even when 30% of the drugs are excluded from our KG. Although *in silico* validation for computationally identified proteins is necessary, true validity of the identified proteins can only be confirmed through experimental validation. For example, gene-knockout studies in animal models allow researchers to assess whether eliminating genes identified by *in silico* models produces a phenotype that mirrors the ADR of interest. Following validation in animal models, clinical studies provide the strongest confirmation of these mechanisms in humans.

One potential and interesting application of this work could be in drug design and repurposing. We identified and showed eight phenotypes (Table S8) where drugs targeting ADR-DP proteins for some phenotypes have indications for diseases with the same phenotype. Extending this idea to other phenotypes, DREAMER can reduce the search space to find relevant protein targets for a particular phenotype. Moreover, DREAMER can

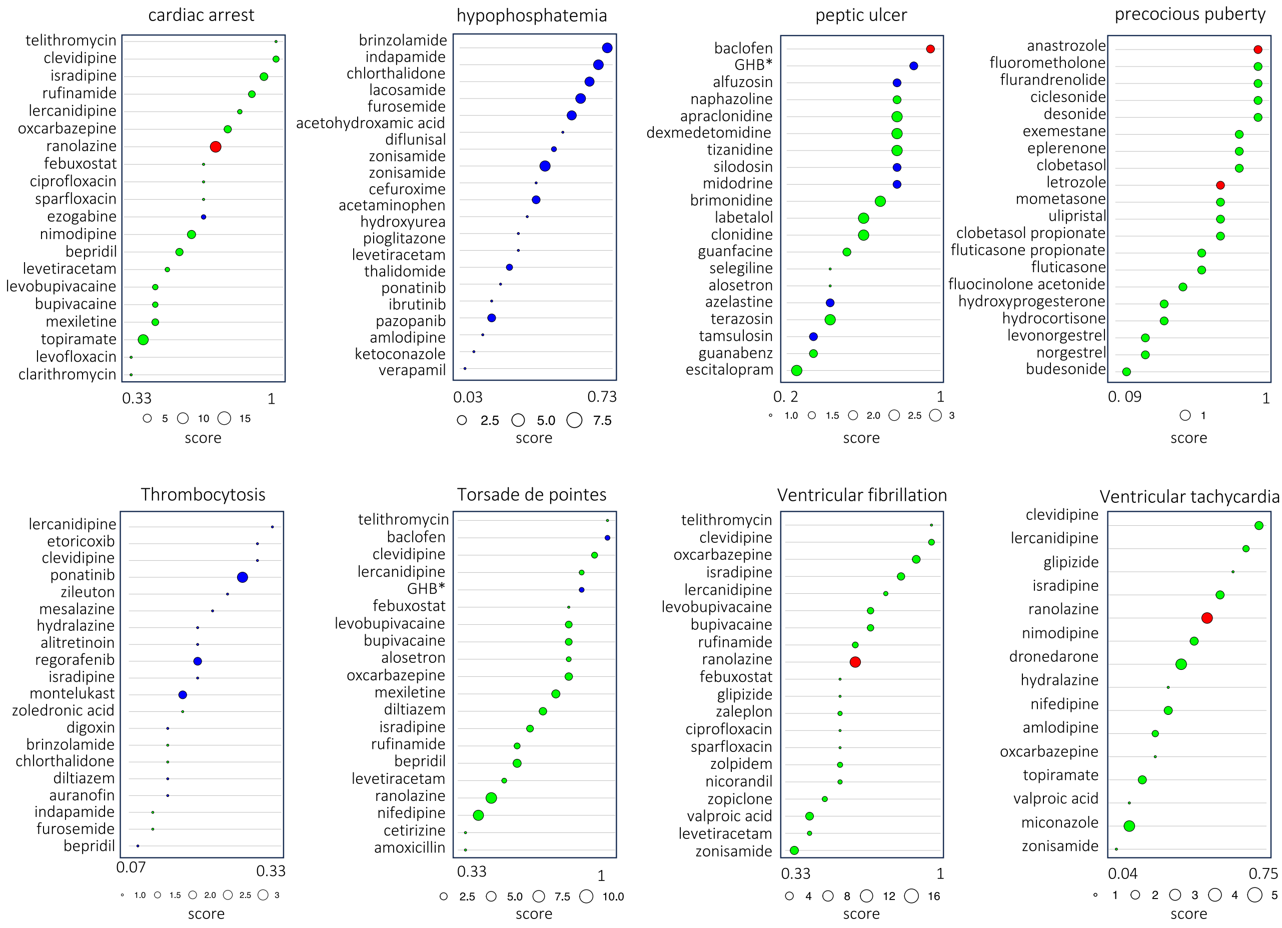

Figure 5. Top-ranked drugs with at least one target in the identified ADR-DP proteins for the phenotypes

Red circles indicate candidate drugs that are found to be under investigation in clinical trials. Green circles indicate candidate drugs that target proteins that are already targeted by indicated drugs. Blue circles indicate candidate drugs whose targets were not already targeted by any indicated drugs. The size of the circles indicates the total number of proteins the candidate drugs target within ADR-DP proteins.: GHB*, gamma-hydroxybutyric acid.

be used for drug off-target prediction. Drugs with a particular ADR are expected to bind to a protein within (or close to) the identified protein sets that govern that ADR (side effect module).[58] The reduced protein space can then be used to infer the potential off-target proteins of drugs using computational methods (e.g., Autodock and Autodock-vina[59,60]) or experimental methods (e.g., based on established physicochemical methods).

Limitations of the study

DREAMER explores the mechanism of phenotypes without considering the specific variations in individual molecular profiles, which are crucial for personalized medicine. To advance our understanding in personalized medicine, one will also require access to individual-specific information. The Food and Drug Administration (FDA) Adverse Event Reporting System[61] provides extensive patient information, including ADRs, drug prescriptions, dosages, and demographic details, which can be leveraged to help elucidate the mechanisms of phenotypes in the context of personalized treatments but ultimately will require molecular-level information with which to generate individual PPIs.[62]

A potential future direction is the investigation of the phenotype mechanism in the context of combination therapy. Drugs can be prescribed as monotherapies or combination therapies,[63] with the latter offering synergistic benefits for complex or multiple disorders but potentially introducing unique ADRs. For instance, in Parkinson's disease, levodopa is prescribed to increase dopamine level and, in combination with that, carbidopa is prescribed to reduce peripheral conversion, reducing the ADRs such as nausea. The TWOSIDES[1] database provides insights into ADRs related to drug combinations and can aid in identifying their mechanisms.[64,65] Additionally, similarities among certain phenotypes can improve the reliability of mechanism identification. Phenotypes can be clustered based on shared drugs and diseases using techniques like biclustering[66] or KG representation learning.[67] In conclusion, DREAMER advances our understanding of ADR and DP mechanisms, offering a valuable tool for improving drug safety, repurposing, and personalized medicine.

RESOURCE AVAILABILITY

Lead contact

Requests for further information and resources should be directed to and will be fulfilled by the lead contact, Farzaneh Firoozbakht (farzaneh.firoozbakht@uni-hamburg.de).

Materials availability

This study did not generate new unique reagents.

Data and code availability

- ADR-phenotype, drug-ADR, drug-protein, gene-disease, gene-protein, drug-indication, and phenotype-disease links and pre-processed STRING PPI network have been deposited at https://doi.org/10.6084/m9.figshare.28254812. Access to drug-protein links requires a usage license from the DrugBank dataset.
- All original code has been deposited at https://github.com/faren-f/DREAMER and is publicly available at https://doi.org/10.6084/m9.figshare.28254812 as of the date of publication.
- Any additional information required to reanalyze the data reported in this work paper is available from the lead contact upon request.

ACKNOWLEDGMENTS

This research was conducted as part of the DrugSiderAI project and was funded by the German Federal Ministry of Education and Research (BMBF) under grant no. 031L0306B to F.F., O.T., and J.B. It was also supported by NIH grants U01 HG007691, R01 HL155107, R01 HL155096, and R01HL166137 to J.L.; AHA grants 957729 and 24MERIT1185447 to J.L.; EU grant Horizon-Health2021 101057619 to J.B. and J.L.; the Swiss State Secretariat for Education, Research and Innovation (SERI) under grant no. 22.00115 to J.B.; Lundbeckfonden under grant no. R347-2020-2454 to M.L.E.; the German Federal Ministry of Education and Research under grant no. 031L0310A to F.F., M.L.E., Z.C., O.T., and J.B.; and the European Union under grant no. 101057619 to F.F., O.T., and J.B. Views and opinions expressed are, however, those of the authors only and do not necessarily reflect those of the European Union or European Health and Digital Executive Agency (HADEA). Neither the European Union nor the granting authority can be held responsible for them. We thank Andreas Maier, Christiane Ehrt, and Olga Zolotareva for their assistance and valuable comments on this manuscript.

AUTHOR CONTRIBUTIONS

Conceptualization, F.F., J.L., O.T., and J.B.; methodology, F.F.; software, F.F.; validation, M.L.E., F.F., D.E.H., and J.L.; formal analysis, F.F.; writing – original draft, F.F.; writing – review & editing, F.F., M.L.E., D.E.H., R.-S.W., Z.C., M.R., J.L., O.T., and J.B.; visualization, F.F. and M.L.E.; supervision, F.F., J.L., O.T., and J.B.; funding acquisition, J.B.

DECLARATION OF INTERESTS

The authors declare no competing interests.

STAR★METHODS

Detailed methods are provided in the online version of this paper and include the following:

- KEY RESOURCES TABLE
- METHOD DETAILS
  - ○ Dataset and knowledge graph construction
  - ○ Overview of the DREAMER
  - ○ Network diffusion
  - ○ Baseline method
- QUANTIFICATION AND STATISTICAL ANALYSIS
  - ○ Hypergeometric test
  - ○ Permutation test

SUPPLEMENTAL INFORMATION

Supplemental information can be found online at https://doi.org/10.1016/j.crmeth.2025.100990.

Received: August 19, 2024
Revised: November 23, 2024
Accepted: January 29, 2025
Published: February 14, 2025

REFERENCES

1. Tatonetti, N.P. (2012). Data-driven Detection, Prediction, and Validation of Drug-Drug Interactions (Stanford University).
2. Kuhn, M., Campillos, M., Letunic, I., Jensen, L.J., and Bork, P. (2010). A side effect resource to capture phenotypic effects of drugs. Mol. Syst. Biol. *6*, 343.
3. Bowes, J., Brown, A.J., Hamon, J., Jarolimek, W., Sridhar, A., Waldron, G., and Whitebread, S. (2012). Reducing safety-related drug attrition: the use of in vitro pharmacological profiling. Nat. Rev. Drug Discov. *11*, 909–922.
4. Garon, S.L., Pavlos, R.K., White, K.D., Brown, N.J., Stone, C.A., Jr., and Phillips, E.J. (2017). Pharmacogenomics of off-target adverse drug reactions. Br. J. Clin. Pharmacol. *83*, 1896–1911.
5. Pirmohamed, M., Breckenridge, A.M., Kitteringham, N.R., and Park, B.K. (1998). Adverse drug reactions. BMJ *316*, 1295–1298.
6. Lounkine, E., Keiser, M.J., Whitebread, S., Mikhailov, D., Hamon, J., Jenkins, J.L., Lavan, P., Weber, E., Doak, A.K., Côté, S., et al. (2012). Large-scale prediction and testing of drug activity on side-effect targets. Nature *486*, 361–367.
7. Wallach, I., Jaitly, N., and Lilien, R. (2010). A structure-based approach for mapping adverse drug reactions to the perturbation of underlying biological pathways. PLoS One *5*, e12063.
8. Mizutani, S., Pauwels, E., Stoven, V., Goto, S., and Yamanishi, Y. (2012). Relating drug–protein interaction network with drug side effects. Bioinformatics *28*, i522–i528.
9. Kuhn, M., Al Banchaabouchi, M., Campillos, M., Jensen, L.J., Gross, C., Gavin, A.-C., and Bork, P. (2013). Systematic identification of proteins that elicit drug side effects. Mol. Syst. Biol. *9*, 663.
10. Lim, H., Poleksic, A., and Xie, L. (2018). Exploring Landscape of Drug-Target-Pathway-Side Effect Associations. AMIA Jt. Summits Transl. Sci. Proc. *2017*, 132–141.
11. Park, J., Lee, S., Kim, K., Jung, J., and Lee, D. (2023). Large-scale prediction of adverse drug reactions-related proteins with network embedding. Bioinformatics *39*, btac843. https://doi.org/10.1093/bioinformatics/btac843.
12. Carss, K.J., Deaton, A.M., Del Rio-Espinola, A., Diogo, D., Fielden, M., Kulkarni, D.A., Moggs, J., Newham, P., Nelson, M.R., Sistare, F.D., et al. (2022). Using human genetics to improve safety assessment of therapeutics. Nat. Rev. Drug Discov. *22*, 145–162.
13. Nguyen, P.A., Born, D.A., Deaton, A.M., Nioi, P., and Ward, L.D. (2019). Author Correction: Phenotypes associated with genes encoding drug targets are predictive of clinical trial side effects. Nat. Commun. *10*, 2132.
14. Pushpakom, S., Iorio, F., Eyers, P.A., Escott, K.J., Hopper, S., Wells, A., Doig, A., Guilliams, T., Latimer, J., McNamee, C., et al. (2019). Drug repurposing: progress, challenges and recommendations. Nat. Rev. Drug Discov. *18*, 41–58.
15. Rubin, D.L., Moreira, D.A., Kanjamala, P.P., and Musen, M.A. 2008 BioPortal: A Web Portal to Biomedical Ontologies. https://cdn.aaai.org/Symposia/Spring/2008/SS-08-07/SS08-07-011.pdf
16. Wishart, D.S., Feunang, Y.D., Guo, A.C., Lo, E.J., Marcu, A., Grant, J.R., Sajed, T., Johnson, D., Li, C., Sayeeda, Z., et al. (2018). DrugBank 5.0: a major update to the DrugBank database for 2018. Nucleic Acids Res. *46*, D1074–D1082.

17. Piñero, J., Bravo, À., Queralt-Rosinach, N., Gutiérrez-Sacristán, A., Deu-Pons, J., Centeno, E., García-García, J., Sanz, F., and Furlong, L.I. (2017). DisGeNET: a comprehensive platform integrating information on human disease-associated genes and variants. Nucleic Acids Res. *45*, D833–D839.

18. von Mering, C., Huynen, M., Jaeggi, D., Schmidt, S., Bork, P., and Snel, B. (2003). STRING: a database of predicted functional associations between proteins. Nucleic Acids Res. *31*, 258–261.

19. Wang, R.-S., and Loscalzo, J. (2021). Network module-based drug repositioning for pulmonary arterial hypertension. CPT Pharmacometrics Syst. Pharmacol. *10*, 994–1005.

20. András, V., Tomek, J., Nagy, N., Virág, L., Passini, E., Rodriguez, B., and Baczkó, I. (2021). Cardiac transmembrane ion channels and action potentials: cellular physiology and arrhythmogenic behavior. Physiol. Rev. *101*, 1083–1176. https://doi.org/10.1152/physrev.00024.2019.

21. Bellocq, C., van Ginneken, A.C.G., Bezzina, C.R., Alders, M., Escande, D., Mannens, M.M.A.M., Baró, I., and Wilde, A.A.M. (2004). Mutation in the KCNQ1 gene leading to the short QT-interval syndrome. Circulation *109*, 2394–2397.

22. Widimsky, P. 2008 Hypokalemia and the heart. https://www.escardio.org/Journals/E-Journal-of-Cardiology-Practice/Volume-7/Hypokalemia-and-the-heart

23. Platonov, P.G., McNitt, S., Polonsky, B., Rosero, S.Z., and Zareba, W. (2019). Atrial Fibrillation in Long QT Syndrome by Genotype. Circ. Arrhythm. Electrophysiol. *12*, e007213.

24. Baroni, D., and Moran, O. (2015). On the multiple roles of the voltage gated sodium channel β1 subunit in genetic diseases. Front. Pharmacol. *6*, 108.

25. Delinière, A., Haddad, C., Herrera-Siklódy, C., Hermida, A., Pruvot, E., Bressieux-Degueldre, S., Millat, G., Janin, A., Hermida, J.-S., Asatryan, B., and Chevalier, P. (2023). Phenotypic Characterization of Timothy Syndrome Caused by the CACNA1C p.Gly402Ser Variant. Circ. Genom. Precis. Med. *16*, 280–282.

26. Borbás, J., Vámos, M., Hategan, L., Hanák, L., Farkas, N., Szakács, Z., Csupor, D., Tél, B., Kupó, P., Csányi, B., et al. (2022). Geno- and phenotypic characteristics and clinical outcomes of CACNA1C gene mutation associated Timothy syndrome, "cardiac only" Timothy syndrome and isolated long QT syndrome 8: A systematic review. Front. Cardiovasc. Med. *9*, 1021009. https://doi.org/10.3389/fcvm.2022.1021009.

27. Acosta-Herrera, M., González-Gay, M.A., Martín, J., and Márquez, A. (2019). Leveraging Genetic Findings for Precision Medicine in Vasculitis. Front. Immunol. *10*, 1796.

28. Pugh, D., Karabayas, M., Basu, N., Cid, M.C., Goel, R., Goodyear, C.S., Grayson, P.C., McAdoo, S.P., Mason, J.C., Owen, C., et al. (2022). Large-vessel vasculitis. Nat. Rev. Dis. Prim. *7*, 93.

29. Wu, D., Nishimura, N., Kuo, V., Fiehn, O., Shahbaz, S., Van Winkle, L., Matsumura, F., and Vogel, C.F.A. (2011). Activation of aryl hydrocarbon receptor induces vascular inflammation and promotes atherosclerosis in apolipoprotein E-/- mice. Arterioscler. Thromb. Vasc. Biol. *31*, 1260–1267.

30. Nakajima, R., Miyagaki, T., Morimura, S., Fukasawa, T., Oka, T., Yoshizaki, A., Sugaya, M., and Sato, S. (2018). Exacerbated Immune Complex-Mediated Vascular Injury in Mice with Heterozygous Deficiency of Aryl Hydrocarbon Receptor through Upregulation of Fcγ Receptor III Expression on Macrophages. J. Invest. Dermatol. *138*, 2195–2204.

31. Tremoulet, A.H. (2015). The role of statins in inflammatory vasculitides. Autoimmunity *48*, 177–180.

32. Motoji, Y., Fukazawa, R., Matsui, R., Nagi-Miura, N., Miyagi, Y., Itoh, Y., and Ishii, Y. (2022). Kawasaki Disease-like Vasculitis Facilitates Atherosclerosis, and Statin Shows a Significant Antiatherosclerosis and Anti-Inflammatory Effect in a Kawasaki Disease Model Mouse. Biomedicines *10*, 1794. https://doi.org/10.3390/biomedicines10081794.

33. Ye, J.Y., Chan, G.C.F., Qiao, L., Lian, Q., Meng, F.Y., Luo, X.Q., Khachigian, L.M., Ma, M., Deng, R., Chen, J.L., et al. (2010). Platelet-derived growth factor enhances platelet recovery in a murine model of radiation-induced thrombocytopenia and reduces apoptosis in megakaryocytes via its receptors and the PI3-k/Akt pathway. Haematologica *95*, 1745–1753.

34. Kandarpa, M., Robinson, D., Wu, Y.-M., Qin, T., Pettit, K., Li, Q., Luker, G., Sartor, M., Chinnaiyan, A., and Talpaz, M. (2024). Broad next generation integrated sequencing of myelofibrosis identifies disease-specific and age-related genomic alterations. Clin. Cancer Res. *30*, 1972–1983. https://doi.org/10.1158/1078-0432.CCR-23-0372.

35. Lu, K., Yang, K., Sun, H., Zhang, Q., Zheng, Q., Xu, K., Chen, J., and Zhou, X. (2023). SympGAN: A systematic knowledge integration system for symptom–gene associations network. Knowl. Base Syst. *276*, 110752.

36. Carvalho-Silva, D., Pierleoni, A., Pignatelli, M., Ong, C., Fumis, L., Karamanis, N., Carmona, M., Faulconbridge, A., Hercules, A., McAuley, E., et al. (2019). Open Targets Platform: new developments and updates two years on. Nucleic Acids Res. *47*, D1056–D1065.

37. Joeres, R., Blumenthal, D.B., and Kalinina, O.V. (2023). DataSAIL: Data Splitting Against Information Leakage. Preprint at bioRxiv. https://doi.org/10.1101/2023.11.15.566305.

38. Ley, K., Laudanna, C., Cybulsky, M.I., and Nourshargh, S. (2007). Getting to the site of inflammation: the leukocyte adhesion cascade updated. Nat. Rev. Immunol. *7*, 678–689.

39. Todd, W.D., Venner, A., Anaclet, C., Broadhurst, R.Y., De Luca, R., Bandaru, S.S., Issokson, L., Hablitz, L.M., Cravetchi, O., Arrigoni, E., et al. (2020). Suprachiasmatic VIP neurons are required for normal circadian rhythmicity and comprised of molecularly distinct subpopulations. Nat. Commun. *11*, 4410.

40. Croft, D., O'Kelly, G., Wu, G., Haw, R., Gillespie, M., Matthews, L., Caudy, M., Garapati, P., Gopinath, G., Jassal, B., et al. (2011). Reactome: a database of reactions, pathways and biological processes. Nucleic Acids Res. *39*, D691–D697.

41. Harris, M.A., Clark, J., Ireland, A., Lomax, J., Ashburner, M., Foulger, R., Eilbeck, K., Lewis, S., Marshall, B., Mungall, C., et al. (2004). The Gene Ontology (GO) database and informatics resource. Nucleic Acids Res. *32*, D258–D261.

42. Berend, K., van Hulsteijn, L.H., and Gans, R.O.B. (2012). Chloride: the queen of electrolytes? Eur. J. Intern. Med. *23*, 203–211.

43. Niemeyer, B.A., Mery, L., Zawar, C., Suckow, A., Monje, F., Pardo, L.A., Stuhmer, W., Flockerzi, V., and Hoth, M. (2001). Ion channels in health and disease. EMBO Rep. *2*, 568–573.

44. Holmes, G.L., and Ben-Ari, Y. (2001). The neurobiology and consequences of epilepsy in the developing brain. Pediatr. Res. *49*, 320–325.

45. Luscher, B., Shen, Q., and Sahir, N. (2011). The GABAergic deficit hypothesis of major depressive disorder. Mol. Psychiatry *16*, 383–406.

46. Gonzalez-Burgos, G., Fish, K.N., and Lewis, D.A. (2011). GABA neuron alterations, cortical circuit dysfunction and cognitive deficits in schizophrenia. Neural Plast. *2011*, 723184.

47. Brambilla, P., Perez, J., Barale, F., Schettini, G., and Soares, J.C. (2003). GABAergic dysfunction in mood disorders. Mol. Psychiatry *8*, 721–737.

48. Horder, J., Petrinovic, M.M., Mendez, M.A., Bruns, A., Takumi, T., Spooren, W., Barker, G.J., Künnecke, B., and Murphy, D.G. (2018). Glutamate and GABA in autism spectrum disorder-a translational magnetic resonance spectroscopy study in man and rodent models. Transl. Psychiatry *8*, 106.

49. Hasler, G., and Northoff, G. (2011). Discovering imaging endophenotypes for major depression. Mol. Psychiatry *16*, 604–619.

50. Yang, R., Walder-Christensen, K.K., Kim, N., Wu, D., Lorenzo, D.N., Badea, A., Jiang, Y.-H., Yin, H.H., Wetsel, W.C., and Bennett, V. (2019). ANK2 autism mutation targeting giant ankyrin-B promotes axon branching and ectopic connectivity. Proc. Natl. Acad. Sci. USA *116*, 15262–15271.

51. Kpaeyeh, J.A., Jr., and Wharton, J.M. (2016). Sotalol. Card. Electrophysiol. Clin. *8*, 437–452.

52. Wharton, J.M., Piccini, J.P., Koren, A., Huse, S., and Ronk, C.J. (2022). Comparative Safety and Effectiveness of Sotalol Versus Dronedarone

After Catheter Ablation for Atrial Fibrillation. J. Am. Heart Assoc. *11*, e020506.

53. Hohnloser, S.H., and Woosley, R.L. (1994). Sotalol. N. Engl. J. Med. *331*, 31–38.

54. Andrade, J.G., and Deyell, M.W. (2022). A Role for Ranolazine in the Treatment of Ventricular Arrhythmias? JACC. Clin. Electrophysiol. *8*, 763–765.

55. Barabási, A.-L., Gulbahce, N., and Loscalzo, J. (2011). Network medicine: a network-based approach to human disease. Nat. Rev. Genet. *12*, 56–68.

56. Musa, A., Tripathi, S., Dehmer, M., Yli-Harja, O., Kauffman, S.A., and Emmert-Streib, F. (2019). Systems pharmacogenomic landscape of drug similarities from LINCS data: Drug association networks. Sci. Rep. *9*, 7849.

57. Berger, S.I., and Iyengar, R. (2009). Network analyses in systems pharmacology. Bioinformatics *25*, 2466–2472.

58. Paci, P., Fiscon, G., Conte, F., Wang, R.-S., Handy, D.E., Farina, L., and Loscalzo, J. (2022). Comprehensive network medicine-based drug repositioning via integration of therapeutic efficacy and side effects. NPJ Syst. Biol. Appl. *8*, 12.

59. Trott, O., and Olson, A.J. (2010). AutoDock Vina: improving the speed and accuracy of docking with a new scoring function, efficient optimization, and multithreading. J. Comput. Chem. *31*, 455–461.

60. Vieira, T.F., and Sousa, S.F. (2019). Comparing AutoDock and Vina in Ligand/Decoy Discrimination for Virtual Screening. Appl. Sci. *9*, 4538.

61. Center for Drug Evaluation, and Research (2021). FDA Adverse Event Reporting System (FAERS) Public Dashboard (U.S. Food and Drug Administration). https://www.fda.gov/drugs/questions-and-answers-fdas-adverse-event-reporting-system-faers/fda-adverse-event-reporting-system-faers-public-dashboard.

62. Maron, B.A., Wang, R.-S., Shevtsov, S., Drakos, S.G., Arons, E., Wever-Pinzon, O., Huggins, G.S., Samokhin, A.O., Oldham, W.M., Aguib, Y., et al. (2021). Individualized interactomes for network-based precision medicine in hypertrophic cardiomyopathy with implications for other clinical pathophenotypes. Nat. Commun. *12*, 873.

63. Maciejewski, M., Lounkine, E., Whitebread, S., Farmer, P., DuMouchel, W., Shoichet, B.K., and Urban, L. (2017). Reverse translation of adverse event reports paves the way for de-risking preclinical off-targets. eLife *6*, e25818. https://doi.org/10.7554/eLife.25818.

64. Zitnik, M., Agrawal, M., and Leskovec, J. (2018). Modeling polypharmacy side effects with graph convolutional networks. Bioinformatics *34*, i457–i466.

65. Deac, A., Huang, Y.-H., Veličković, P., Liò, P., and Tang, J. (2019). Drug-Drug Adverse Effect Prediction with Graph Co-Attention. Preprint at arXiv. https://doi.org/10.48550/arXiv.1905.00534.

66. Hartung, M., Maier, A., Delgado-Chaves, F., Burankova, Y., Isaeva, O.I., Patroni, F.M. de S., He, D., Shannon, C., Kaufmann, K., Lohmann, J., et al. (2024). UnPaSt: unsupervised patient stratification by differentially expressed biclusters in omics data. Preprint at arXiv. https://doi.org/10.48550/arXiv.2408.00200.

67. Hamilton, W.L. (2020). Graph Representation Learning (Morgan & Claypool Publishers).

68. Kuhn, M., Letunic, I., Jensen, L.J., and Bork, P. (2016). The SIDER database of drugs and side effects. Nucleic Acids Res. *44*, D1075–D1079.

69. Köhler, S., Vasilevsky, N.A., Engelstad, M., Foster, E., McMurry, J., Aymé, S., Baynam, G., Bello, S.M., Boerkoel, C.F., Boycott, K.M., et al. (2017). The Human Phenotype Ontology in 2017. Nucleic Acids Res. *45*, D865–D876.

70. Sadegh, S., Skelton, J., Anastasi, E., Bernett, J., Blumenthal, D.B., Galindez, G., Salgado-Albarrán, M., Lazareva, O., Flanagan, K., Cockell, S., et al. (2021). Network medicine for disease module identification and drug repurposing with the NeDRex platform. Nat. Commun. *12*, 6848.

71. Brin, S., and Page, L. (1998). The anatomy of a large-scale hypertextual Web search engine. Comput. Netw. ISDN Syst. *30*, 107–117.

72. Hofree, M., Shen, J.P., Carter, H., Gross, A., and Ideker, T. (2013). Network-based stratification of tumor mutations. Nat. Methods *10*, 1108–1115.

CellPress
OPEN ACCESS

STAR★METHODS

KEY RESOURCES TABLE

REAGENT or RESOURCE	SOURCE	IDENTIFIER
Deposited data		
Analyzed data	This paper	https://doi.org/10.6084/m9.figshare.28254812
SIDER 4.1: Side Effect Resource	Kuhn et al.[68]	http://sideeffects.embl.de/
The Human Phenotype Ontology	Köhler et al.[69]	https://hpo.jax.org/
BioPortal	Rubin et al.[15]	https://bioportal.bioontology.org/
DrugBank	Wishart et al.[16]	https://go.drugbank.com/
DisGeNET	Piñero et al.[17]	https://disgenet.com/
STRING	von Mering et al.[18]	https://string-db.org/
Physical protein-protein interaction	Wang et al.[19]	https://github.com/bwh784/PAHdrugs
Software and algorithms		
Code	This paper	https://doi.org/10.6084/m9.figshare.28254812
R programming environment	https://www.r-project.org/	R version 4.3.2
RStudio (IDE for R)	https://posit.co/products/open-source/rstudio/	Version 2023.09.1 + 494

METHOD DETAILS

Dataset and knowledge graph construction

Our KG is composed of different node types of drugs, diseases, proteins, ADRs, and DPs. To construct this KG, we utilized data from different sources along and applied several preprocessing steps, as described in the following (Figure 2).

Drug-ADR association

To obtain the Drug-ADR association, we used the latest release of the SIDER database (SIDER 4).[68] SIDER is a common mono-pharmacy ADR benchmark and publicly available database that compiles information on post-marketing drug-ADR associations extracted from several public sources, including the US Food and Drug Administration (FDA). ADRs are mapped to UMLS and MedDRA terms in the SIDER database. We considered more specific phenotype MedDRA identifiers (low-level term), where MedDRA (Medical Dictionary for Regulatory Activities) is a medical terminology database used to standardize reporting of ADRs and other regulatory activities. We excluded links between ADRs and drugs that have indications equivalent to the ADRs in our datasets, which might be due to rare and unusual conditions or false positive reports. For example, metoprolol is a drug used to treat angina pectoris and hypertension while hypertension is listed as one of its ADRs. Additionally, we excluded ADRs linked to a large number of drugs (>50), such as headache, nausea, skin rash, and diarrhea, as very common ADRs (Figure S1A).

Disease-phenotype association

Disease-DP associations were obtained from the human phenotype ontology (HPO) database.[69] For DPs, we used the HPO identifiers, a standardized vocabulary of phenotypic abnormalities observed in human disease, and removed DPs linked to a large number of diseases (>100), such as seizure, hypotonia, and microcephaly, as very common DPs (Figure S1B).

ADR-DP association

The ADR-DPs associations are collected from the Bioportal database,[15] which reports 1,240 such relations. Among them, we preserved only ADRs associated with at least one drug that has a known protein target; and DP associated with at least one disease linked to a known gene that codes for a protein present in our dataset. In addition, in some cases, a DP (with a unique HPO ID) is linked to several ADRs (with their MedDRA IDs), among which we only selected one of them by random. This process resulted in a total of 649 ADR-phenotype associations.

Drug-target association

Drug-target associations are obtained from the DrugBank database with a premium license.[16] We excluded drugs that had no known associations with proteins from our KG.In addition, drug-target associations were excluded if the drug was not included in the SIDER database.

Disease-gene association

Disease-gene associations were obtained from the DisGeNET database.[17] Here we used the expert-curated resource available in DisGeNET. We excluded disaeses that had no known associations with proteins.

Protein-protein association

Protein-protein associations were obtained from the STRING database,[18] an extensive resource that aggregates both experimental and predicted interaction data. We only considered associations with a high confidence score (above 800) to ensure the reliability of the inferred protein interaction networks in our analysis. While STRING encompasses a wide variety of interactions, including both direct (first order) and indirect (higher order) PPIs, we further analyzed the results using only physical interactions as the basis for constructing the PPI network,[19] which is more specific. The physical PPI network was downloaded from https://github.com/bwh784/PAHdrugs. Table S1 shows a summary of node types and edge types, their number, and the databases that are used to extract this information.

All edges except for STRING and physical PPI networks are obtained from the NeDRexDB platform,[70] which is an integrative and up-to-date database for drug repurposing and disease module discovery.

Overview of the DREAMER

To investigate the underlying mechanisms of a phenotype, DREAMER systematically identifies proteins that are highly related to a pair of ADR and DP representing the same phenotype. For a protein to be considered relevant, it should successfully pass three statistical tests.

(1) it must demonstrate significant relevance to the ADR (ADR-related protein);
(2) it must demonstrate significant relevance to the DP (DP-related protein);
(3) there must be a significant overlap in the findings from (1) and (2).

The step-by-step process of our proposed algorithm to identify ADR-DP proteins is as follows.

(1) Identifying ADR-related seed proteins: for each ADRj, we start by listing the associated drugs, followed by listing all protein targets of these drugs. Each protein is then assigned a probability based on the number of its links to the listed drugs, calculated as follows:

$$p_{ADR_j}(protein_i) = \frac{Number\ of\ links\ between\ protein_i\ and\ drugs\ with\ ADR_j}{Number\ of\ all\ links\ between\ drugs\ with\ ADR_j\ and\ their\ targets}$$

Other proteins in the network, which have no links to these drugs, are assigned a probability of zero as the initial condition.

(2) Diffusion from ADR-related seed proteins: using these probabilities as the initial seed vector, we run a network diffusion algorithm, called personalized PageRank (see below) on the PPI network to propagate the signal from protein targets to other proteins in the network. This generates a score vector $s_{ADR_j}(protein_i)$, representing the score of each protein i in relation to ADR_j.
(3) Identifying ADR-related proteins: to assess the significance of each protein's score obtained in step 2, we perform a permutation test by shuffling the initial vectors 1,000 times, yielding null diffusion scores. A one-sided p-value is calculated for each protein by fitting an exponential distribution to the empirical null distribution and corrected using the Benjamini-Hochberg method. We then selected the proteins with p-values less than 0.05 as ADR-related proteins (Figure 1B).
(4) Identifying DP-related seed proteins: for each DP_j that exhibits the same phenotype as the given ADR_j in step 1, we start by listing its associated diseases. For these diseases we list their associated genes and then their coded proteins. Similar to step 2, each protein is then assigned a probability based on the number of its links to the listed diseases, calculated as follows:

$$p_{DP_j}(protein_i) = \frac{Number\ of\ links\ between\ protein_i\ and\ diseases\ with\ DP_j}{Number\ of\ all\ links\ between\ diseases\ with\ DP_j\ and\ associated\ proteins}$$

Again, proteins without any link to these diseases are assigned a probability of zero as the initial condition.

(5) Diffusion from DP-related seed proteins: using these probabilities as the initial seed vector, we run the network diffusion algorithm on the PPI network that results in score vectors $s_{DP_j}(protein_i)$, representing the score of each protein i in DP_j.
(6) Identifying DP-related proteins: to assess the significance of each protein's score identified in step 5, we perform the same permutation test as described in step 3 resulting in p-values calculated for proteins. We then selected the proteins with p-values less than 0.05 as DP-related proteins (Figure 1C).
(7) Identifying ADR-DP related proteins: finally, we select the proteins at the intersection of ADR- and DP-related sets, provided that their overlap is statistically significant ($p < 0.05$, hypergeometric test corrected by BH; Figure 1D).

The step-by-step process of identifying indication-related proteins is as follows.

(1) Identifying indication-related seed proteins: for each ADR_j, we start by listing the associated diseases that are indicated for all drugs linked to ADR_j. Next we list all proteins associated with those diseases. Each protein is then assigned a probability based on the number of its links to the listed diseases, calculated as follows:

$$p'_{ADR_j}(protein_i) = \frac{\text{Number of links between } protein_i \text{ and diseases indicated by all drugs with } ADR_j}{\text{Number of all links between diseases indicated by all drugs with } ADR_j \text{ and their associated proteins}}$$

Other proteins in the network, which have no links to these diseases, are assigned a probability of zero as the initial condition.

(2) Diffusion from indication-related seed proteins: using these probabilities as the initial seed vector, we run a network diffusion algorithm on the PPI network that results in score vectors $s'_{ADR_j}(protein_i)$, representing the score of each protein i in relation to ADR_j through the diseases indicated by drugs related to ADR_j.
(3) Identifying indication-related proteins: to assess the significance of each protein's score, we perform the same permutation test as described in the step 3 above resulting in p-values calculated for proteins. Finally, we selected the proteins with p-values less than 0.05 as indication-related proteins (Figure 1F).

Network diffusion

Network diffusion is performed for each ADR (DP) independently through the PPI network initiated from drug targets (disease associated proteins) as mentioned above. To this end, we use the personalized page rank (PPR - the R package *igraph* v1.5), which is an algorithm developed by google to rank web pages based on their relevance to a specific user or topic.[71] The PPR can capture the flow of information through the network by considering a walker that explores the network by taking steps in different directions starting from initial seed nodes. As a result, the walker visits nodes that are close to the seed nodes (probably being involved in the same mechanism) more often than the rest of the nodes in the network. The iterative formula of PPR can be defined as

$$P_{t+1} = \alpha . A . P_t + (1 - \alpha) . P_0$$

Where P_{t+1} is the diffusion vector after the $t+1$-th iteration, α is damping factor set to 0.7 as suggested by[72] for the STRING network. A is the transition probability matrix or the adjacency matrix in the PPI. Finally, P_0 is personalization vector or preference vector, which is served as the initial score.

Baseline method

To assess the efficiency of the diffusion-based algorithm, we considered a method used in Kuhn et al. as the baseline method.[9] This method recognizes two protein sets for each phenotype, one for the ADR and the other for DP. We used the same KG (Figure S2), including the same ADRs and DPs for the baseline method (i.e., without the diffusion process). This method assigns a protein to an ADR (or DP) if there is a significant overlap between the set of drugs (or diseases) linked to that protein and the set of drugs (or diseases) linked to the given ADR (DP). To check for the significance of the overlap, we used a hypergeometric test. We refer to these protein sets as baseline-ADR proteins and baseline-DP proteins for ADRs and DPs, respectively.

QUANTIFICATION AND STATISTICAL ANALYSIS

All statistical analyses were performed using R version 4.3.2 described in the following.

Hypergeometric test

Used to assess the significance of overlap between protein sets (e.g., ADR-related and DP-related proteins). p values were corrected using the Benjamini-Hochberg method, with a significance threshold of $\alpha = 0.05$.

Permutation test

Applied to assess the significance of protein scores from network diffusion. For each protein, 1,000 permutations of the seed vectors were used to generate null distributions, and p values were computed by fitting an exponential distribution to the null scores. Corrected p values Benjamini-Hochberg below 0.05 were considered significant.

Cell Reports Methods, Volume *5*

Supplemental information

Exploring common mechanisms of adverse drug reactions and disease phenotypes through network-based analysis

Farzaneh Firoozbakht, Maria Louise Elkjaer, Diane E. Handy, Rui-Sheng Wang, Zoe Chervontseva, Matthias Rarey, Joseph Loscalzo, Jan Baumbach, and Olga Tsoy

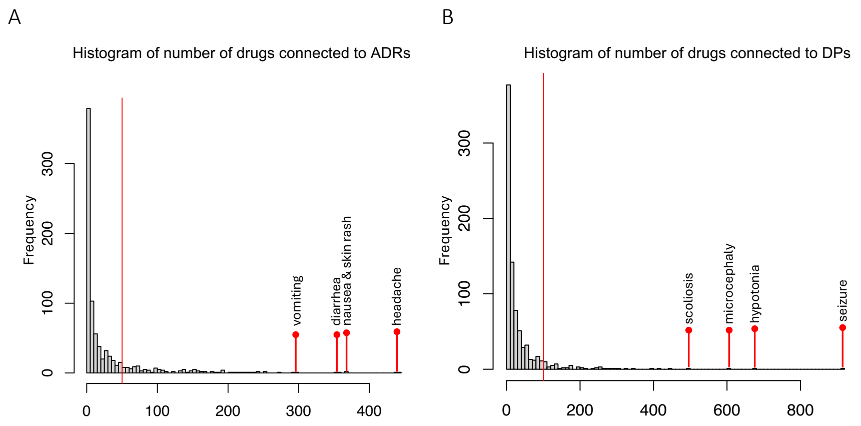

Figure S1. The distribution of the number of drugs and diseases connected to ADRs and DPs, related to STAR Methods.

(A) Number of drugs connected to ADRs. The vertical red line indicates the threshold used to remove ADRs connected to more than 50 drugs.

(B) Number of diseases connected to DPs. The vertical red line indicates the threshold used to remove DPs that are connected to more than100 diseases.

Table S1. The summary statistics of nodes and edges in our knowledge graph, related to STAR Methods.

Node/edge type	Initial count	# nodes &edges after preprocessing	Database
ADR	10368	649	MedDRA
DP	16874	649	HPO
Drug	6177	465	Drugbank
Disease	22825	2553	Mondo Disease Ontology
Protein	19385	12694	UniProt
ADR-[is the same as]-Phenotype	1200	649	BioPortal
ADR-[is reported for]-Drug	217384	6358	Sider
Drug-[has target]-Protein	26537	5185	DrugBank
Phenotype-[is reported for]- Disease	227812	9528	HPO
Gene-[associated with]-Disease	26841	6652	DisGeNET
Gene-[encoded by]-Protein	33246	3417	UniProt
Protein-[interacts with]-Protein	11938499	127767	STRING
Protein-[interacts with]-Protein	–	346535	Physical network

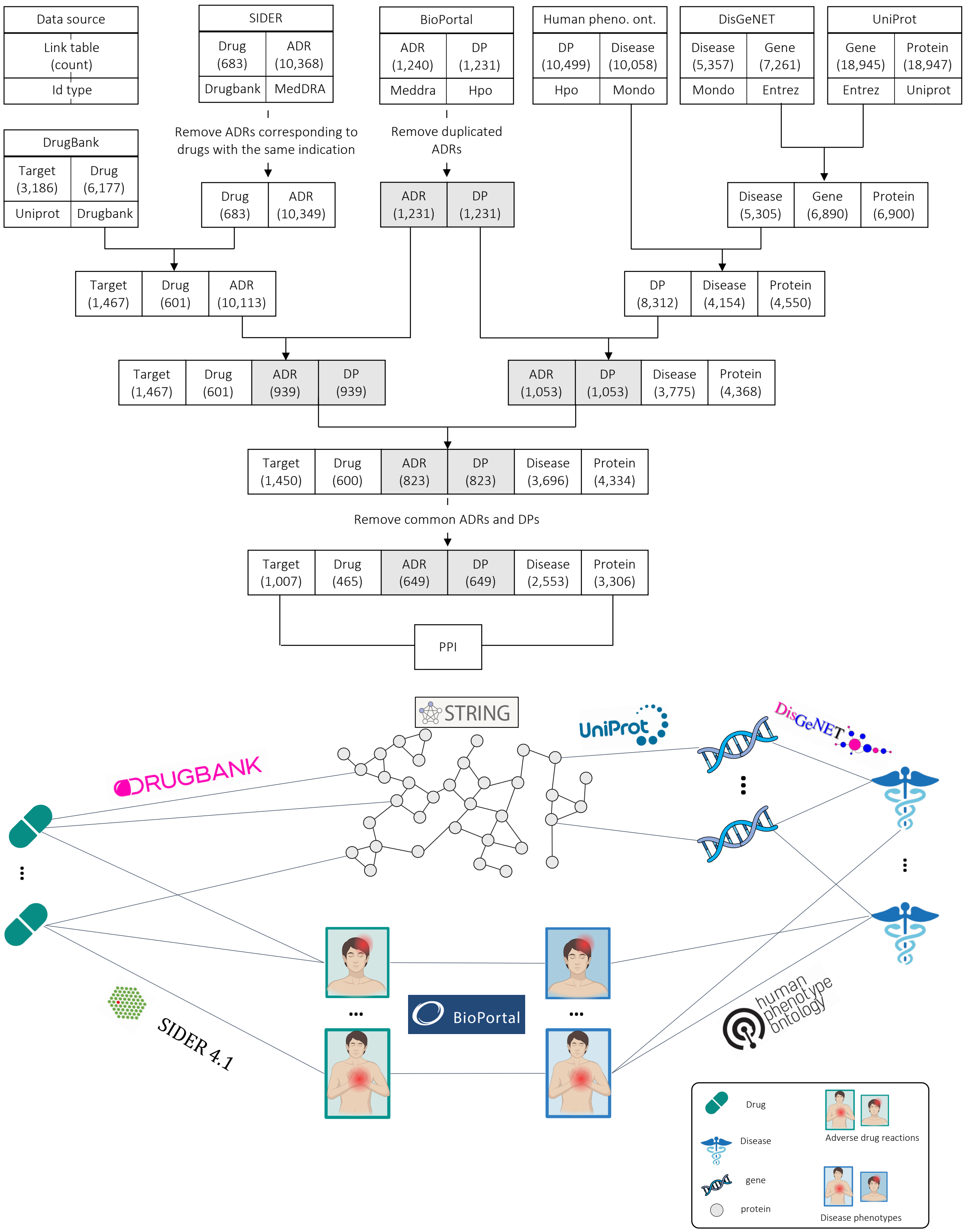

Figure S2. An overview of our knowledge graph, data sources and pre-processing, related to STAR Methods.

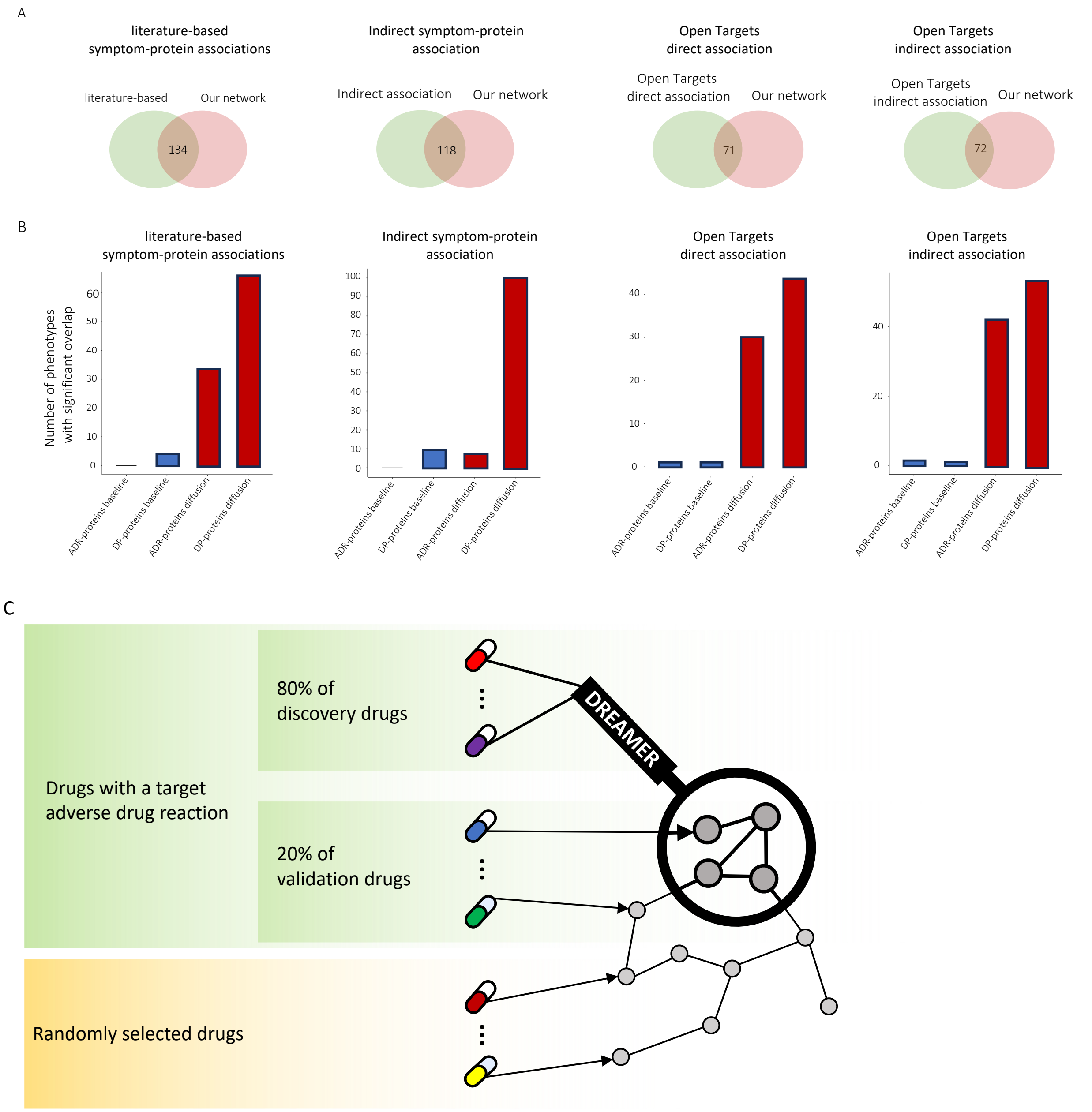

Figure S3. Reliability assessment of the identified protein set using the network diffusion algorithm on the physical protein-protein interaction network and schematic representing the hold-out validation analysis, related to Figure 2.

(A) Number of shared phenotypes with our constructed KG and known databases.

(B) Comparison of methods using literature-based dataset, indirect association based on disease-phenotypes equivalent terms, direct associations in Open Targets dataset, indirect associations in Open Targets dataset.

(C) Drugs with a particular ADR are split into a discovery set (80%) and a validation set (20%). The discovery drugs are used to identify ADR-DP proteins by the DREAMER pipeline. Our analysis shows that these ADR-DP proteins are closer to the validation drugs compared to randomly selected drugs that do not have the given ADR.

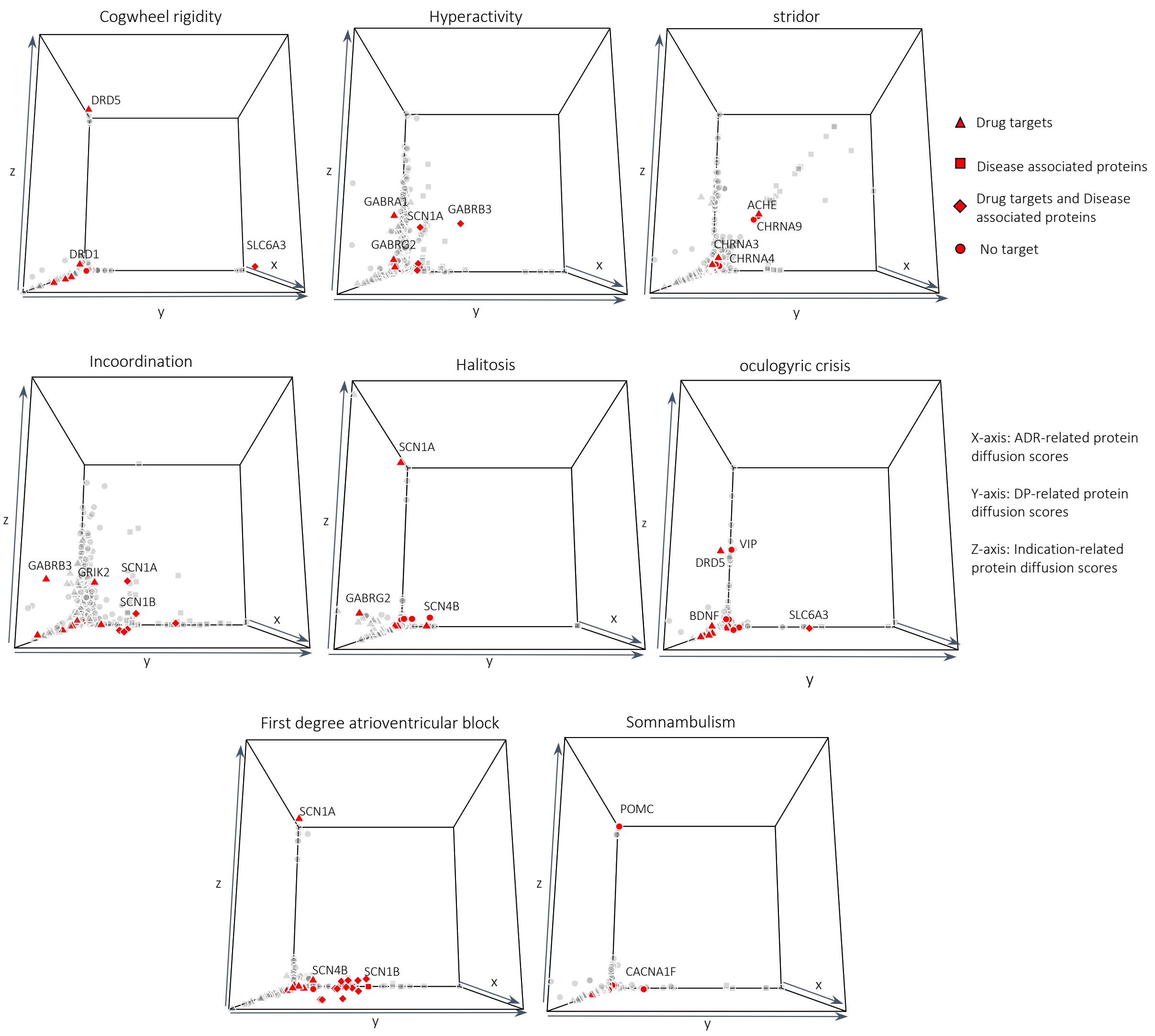

Figure S4. 3D diffusion maps, related to Figure 3. x, y, and z-axis represent the diffusion scores of proteins from drug-targets, disease-proteins, and drug indication-proteins, for cogwheel rigidity, hyperactivity, stridor, incoordination, halitosis, oculogyric crisis, first degree atrioventricular block, somnambulism. The ADR-DP proteins are indicated as red points.

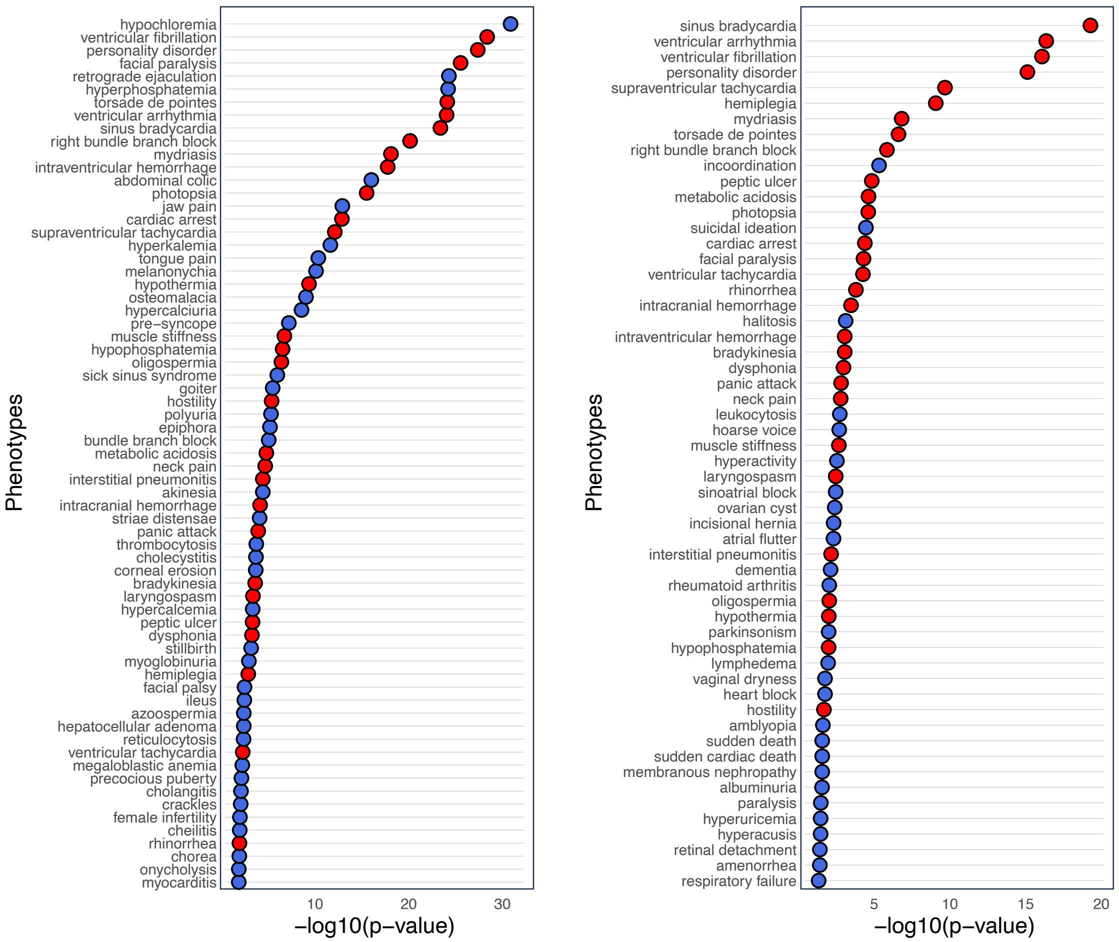

Figure S5. The ranked list of phenotypes based on the significance of their ADR-DP proteins, related to Figure 3. *Left*: for STRING network; *right*: for the physical PPI network. Phenotypes highlighted in “red” circles indicate those for which we identified the mechanism using an analysis based on either the STRING network or the physical PPI network.
